# Supplementary material for: Creation of a point-of-care therapeutics sensor using protein engineering, electrochemical sensing and electronic integration
Source: Nat Commun. 2024 Feb 24;15:1689. doi: 10.1038/s41467-024-45789-9 (PMC11258353; doi:10.1038/s41467-024-45789-9)
Supplement: Supplementary file 1 — Supplementary Information [file 41467_2024_45789_MOESM1_ESM.docx]

**Creation of a Point-of-Care Therapeutics Sensor Using Protein Engineering, Electrochemical Sensing and Electronic Integration**

**Authors:** Rong Cai^*1^, Chiagoziem Ngwadom^1^, Ravindra Saxena^2,3^, Jayashree Soman^1^, Chase Bruggeman^4^, David P. Hickey^4^, Rafael Verduzco^3^, Caroline M. Ajo-Franklin*^1^

**Context**

1. Engineering a switchable GDH using saturated domain-insertion profiling 1

1.1 LBD-GDH library construction 1

1.2 Screening the LBD-GDH library 4

1.3 The rationale for designing GDH-5E^+^ 7

2. Characterization of allosteric LBD-GDH 9

2.1 LBD-GDH expression and purification 9

2.2 LBD-GDH characterization 11

3. Creating a 4-HT amperometric sensor with GDH-5E^+^ 15

3.1 GDH-5E^+^ electrode preparation 15

3.2 Cyclic voltammetry 16

3.3 Amperometric i-t curves 16

3.4 Electrochemistry with human blood sample 17

4. Creating a self-powered sensor 20

4.1 Laccase electrode preparation 20

4.2 Glucose/O_2_ enzymatic fuel cell (EFC) 21

4.3 A self-powered 4-HT sensor 21

5. Coupling EFC with OECT 23

5.1 OECT fabrication 23

5.2 EFC power the gate of OECT 24

5.3 Mathematical model of a self-powered sensor coupled to OECT 25

6. Supplementary References 27

**1. Engineering a switchable GDH using saturated domain-insertion profiling**

**1.1 LBD-GDH library construction.**

We used the Saturated Programmable Insertion Engineering (SPINE) algorithm (*1*) to design and generate a vector library that expresses GDH with LBD inserted across the open reading frame. In silico, the full length of GDH was broken into eight tiles tailing with BsmbI sites. Each oligo (< 300 base pairs) contained a genetic handle, 24 base pairs of nucleotides encoding linkers and BsaI restriction sites. These oligos were microarray-synthesized and amplified as eight oligo pools. Each pool joined its backbone in parallel, then pooled together as the intermediate library. Lastly, the LBD sequence replaced the genetic handle through BsaI-mediated Golden Gate cloning, resulting in the LBD inserted GDH library (Fig. S1).


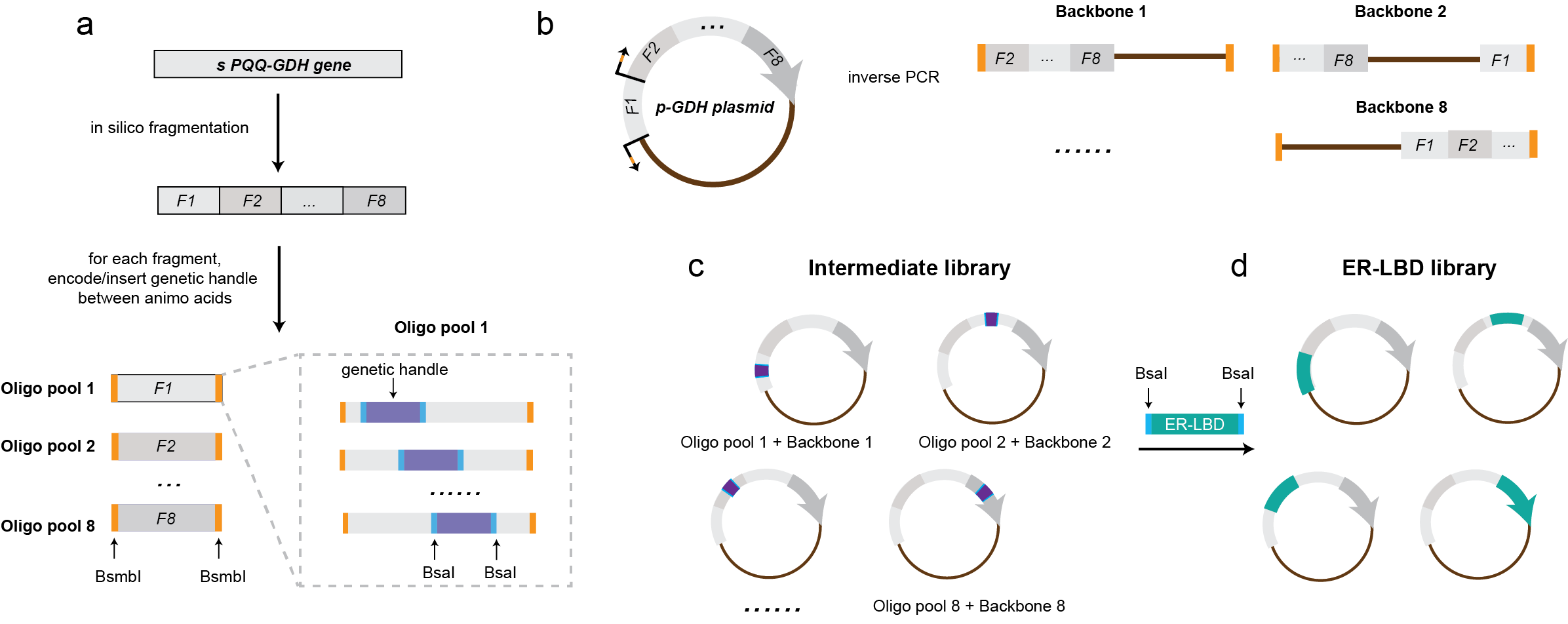


**Figure S1**. **SPINE workflow.** (a) In silico GDH fragmentation and oligo pool design. (b) Inverse PCR preparing backbones. (c) Preparing intermediate library with genetic handle. (d) Replacing genetic handle with ligand binding-domain of estrogen receptor.

**1.1.1 GDH oligo pools in silico design.** Python-GDH plasmid (without BsaI or BsmbI recognition sites) were prepared in FASTA format. The gene sequence was then submitted to the custom algorithm (https://github.com/schmidt-lab/SPINE). This program generates oligo sequences, their corresponding primers for amplification, and the target backbone primers for inverse PCR.

**1.1.2 Oligo pools amplification.** The silico-designed gene pool, which contains 456 oligos with a genetic handle inserted across GDH, was synthesized by Twist Bioscience and received as ~ 80 ng of lyophilized DNA. This DNA was resuspended in TE buffer with a final concentration of 20 fmol/µL. The eight oligo pools were PCR amplified with their corresponding primers using Q5 hot start polymerase (NEB) in 50 µL reactions with 1 µL template for 20 cycles. The entire PCR reaction was run on 3 % agarose gels, visualized with Gel Green (Bio-Rad). The brightest bands were cut for gel purification (Qiagen).

**1.1.3. Combining oligo pool with target gene backbone.** We first prepared eight linear backbones franked by complementary BsmBI sites to their corresponding oligo pools by PCR using Q5 hot start polymerase and python-GDH plasmid as the template. PCR products were run on 1.2 % agarose gels, visualized, and gel purified. The backbone and oligo pools were combined using the Golden Gate assembly kit (BsmBI-v2, from NEB) in a 20 µL reaction with a 2:1 ratio (vector: inserts) at 42 ^◦^C for one hour. The products were directly transformed into *E. coli* DH5α chemically competent cells (NEB) according to the manufacturer’s instructions. The transformed cells were grown overnight at 37 ^◦^C in 2 mL LB with 50 µg/mL kanamycin shaking at 250 rpm. The intermediate library DNA was isolated by miniprep (Qiagen). A small subset of the transformed cells was diluted 100 times and plated on LB with kanamycin agar plates. The CFU of the eight sub-libraries ranged from 1200 to 5200, corresponding to > 99.99 % coverage. The Sanger sequencing confirmed that nearly 80 % of the colonies were perfect variants (Table S1). All eight libraries were pooled together at an equimolar ratio, resulting in the intermediate library with the genetic handle crossing GDH.

**Table S1**. **Sanger sequencing of libraries**


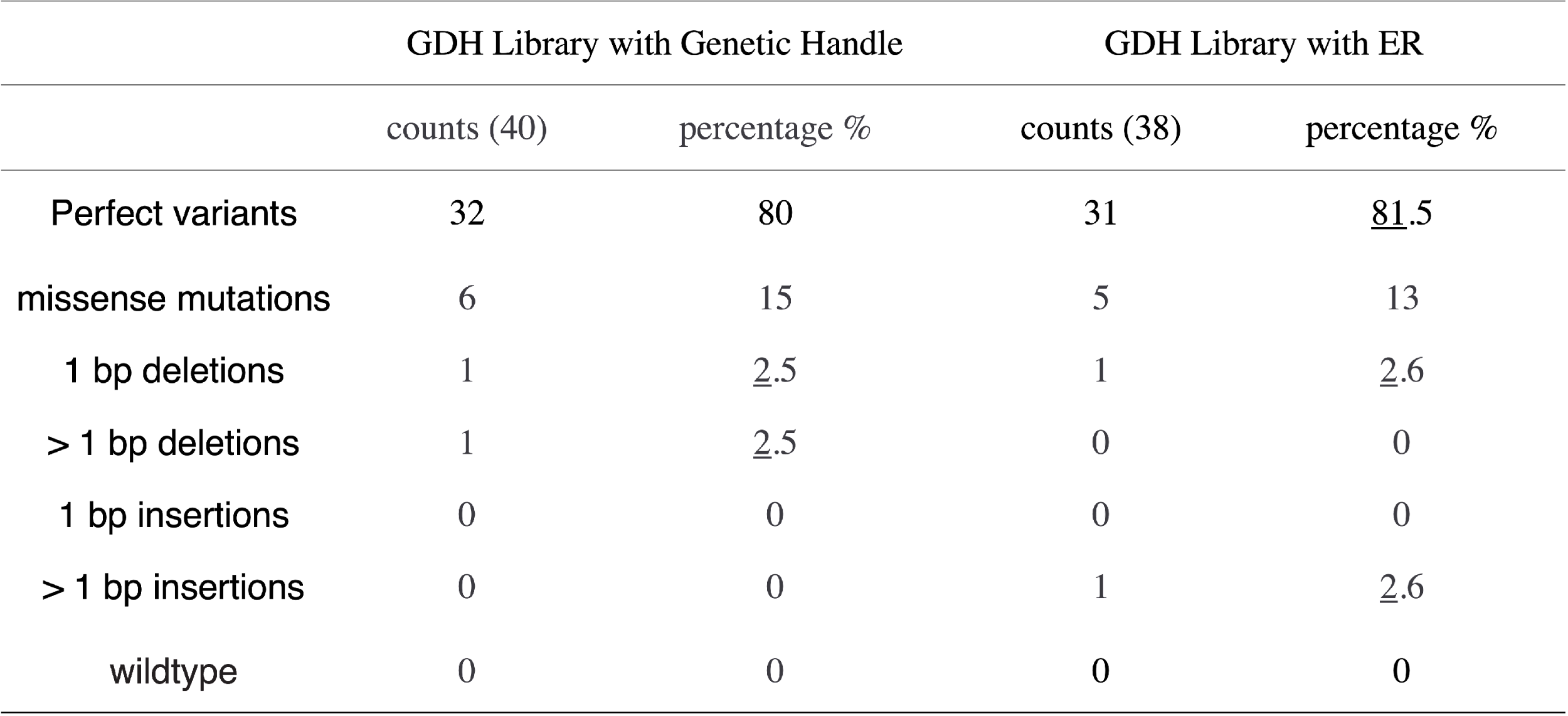


**1.1.4. Replacing the genetic handle with ligand binding domain of estrogen receptor (LBD).** The LBD was a kind gift from Dr. Jonathan J. Silberg (Rice University). BsaI sites complementary to those in the inserted genetic handle were added to LBD by PCR using Q5 hot start polymerase. To limit the background of the GDH with the genetic handle, we first digested 500 ng intermediate library plasmids with BsaI-HFv2 (NEB) for one hour at 37 ^◦^C. After gel extraction, 80 ng of linearized vectors were recycled from 1.2 % agarose gel. The linear vectors were assembled with 23 ng LBD in a 20 µL reaction by Golden Gate assembly kit (BsaI-HFv2, from NEB) at 37 ^◦^C for one hour. The product was directly transformed into *E. coli* NEB-10 beta competent cells. Cells were grown overnight at 37 ^◦^C in 5 mL LB with 50 µg/mL kanamycin shaking at 250 rpm. The LBD-GDH library DNA was isolated by miniprep (Qiagen). This step yielded > 9200 colonies with 81.5 % perfect variants (Table S1).

**1.1.5. Deep sequencing of LBD-GDH library.** The plasmids from the final LBD-GDH library were extracted using a Monarch Plasmid Miniprep kit. The product served as the template for specific LBD-GDH amplification with 15 cycles of PCR using the Q5 hot start polymerase. The resulted amplicon was run on 1.2 % agarose gels, purified by gel extraction, and quantified by Quant-iT Picogreen. A total of 1.6 µg of DNA was prepared for deep sequencing using the Illumina MiSeq, 2 x 150 bp configuration by GENEWIZ. Insertion sites were identified from 140,340 raw sequencing reads. Alignments were processed using the DIP-seq pipeline (http://github.com/SavageLab/dipseq) on both forward and reverse reads (Fig. S2).


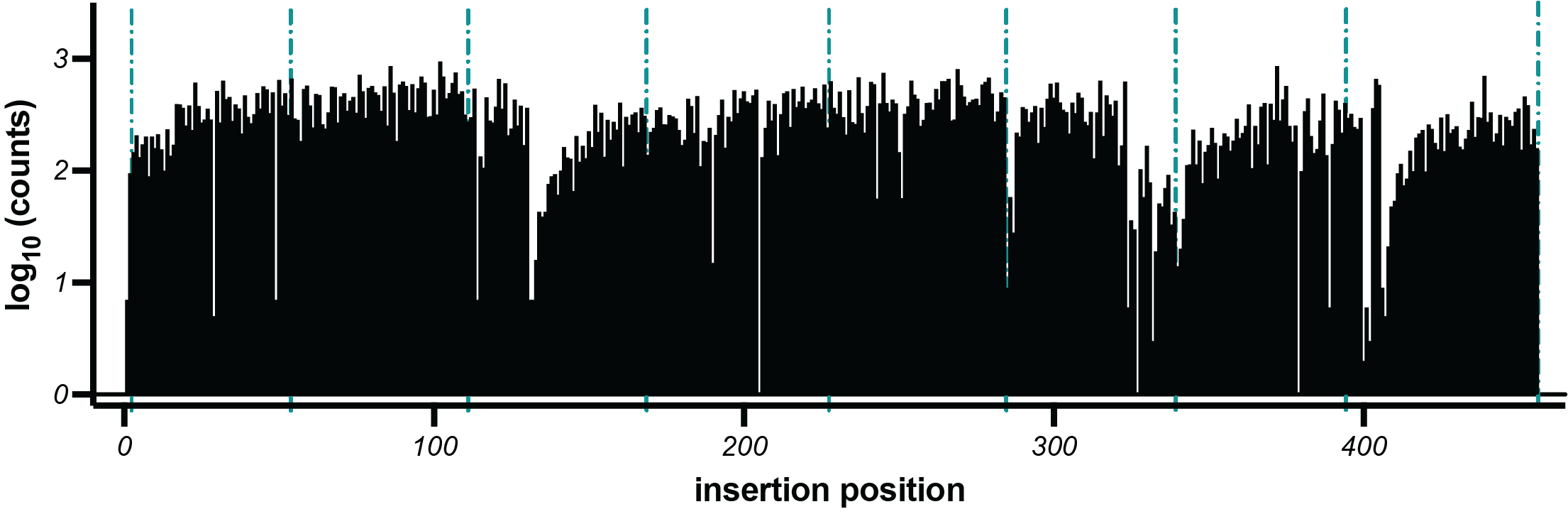


**Figure S2**. **Profile of LBD insertion along GDH.** LBD was inserted after all the 454 amino acids of GDH. The green dashed line indicates the boundary of eight tiles.

**1.2. Screening the LBD-GDH library**

**1.2.1. The probability that the library is complete.** From statistical analysis (Allen et al.(*2*)), the probability $P_{i}$ that a particular sequence in the library occurs, $i$, is calculated as:

$P_{i}=1-{(1-F_{i})}^{T}$ (1)

Where *T* is the number of transformants or screen candidates; $F_{i}$ is the product of the frequency that *i* is expected to be present considering the methods used to create the library and the frequency of “meaningful” library member, like the perfect colony capable of expressing targeting protein.

If the chance of occurrence for each member of the library is equal, the probability of each particular member being present in a library can be calculated as

$P_{c}=\prod_{i=1}^{D} P_{i}$ (2)

Where D is the degeneracy of a library (number of distinct sequences).

Here, we assume each of the 454 sequences has an equal chance of being picked (It is not true because of the bias of the library in Fig. S2). Given the perfect variants in the LBD-GDH library is 81.5% (Table S1), we must screen at least 7249 colonies to complete the entire LBD-GDH library ($P_{c}$ > 99.9) according to the following calculation.

$P_{c}=\left[ 1-{(1-F_{i})}^{T} \right]^{D}$ (3)

$P_{c}=\left[ 1-{(1-\frac{1}{\frac{454}{81.5\%}})}^{T} \right]^{454}$ (4)

**1.2.2. Expression of LBD-GDH**. 136 ng of LBD-GDH library DNA was transformed into 50 µL *E. coli* BL21 chemical component cells (NEB) for protein expression. The transformed cells were diluted to various concentrations and grown on LB agar plates with 50 µg/mL kanamycin overnight at 37 ^◦^C. The following day, single colonies were picked and transferred to 96 deep-well plates in 600 µL of LB medium, complementing with 10 mM CaCl_2_, 1 µM PQQ, 50 µM IPTG and 50 µg/mL kanamycin. The cells grew at 25 ^◦^C with shaking at 60 rpm for 18 hours. The final OD_600_ were 0.4 ~ 0.6.

**1.2.3. GDH activity colorimetric assay**. GDH activity was determined spectrophotometrically at room temperature by following the reduction of dichlorophenolindophenol (DCPIP) at 600 nm, using phenazine methosulfate (PMS) as a primary electron mediator (Fig. S3). A reagent solution containing 45 mL MOPS buffer (10 mM, pH 7), 1 mL DCPIP (20 mg dissolved in 5 mL of H_2_O overnight), 1 mL PMS (45 mg in 5 mL of H_2_O, freshly made and kept in the dark) and 1 mL glucose (1 M in MOPS, mutarotate for overnight) were prepared in each use. GDH activity was correlated to the velocity ($v$) of DCPIP oxidation which was reported as the absorption decreasing at 600 nm over time. The coefficient of variance for whole cells assay was calculated as 11% (Fig. S4). Given the ideal coefficient of variance is 10%,(*3*) our assay provided a reliable screen approach.


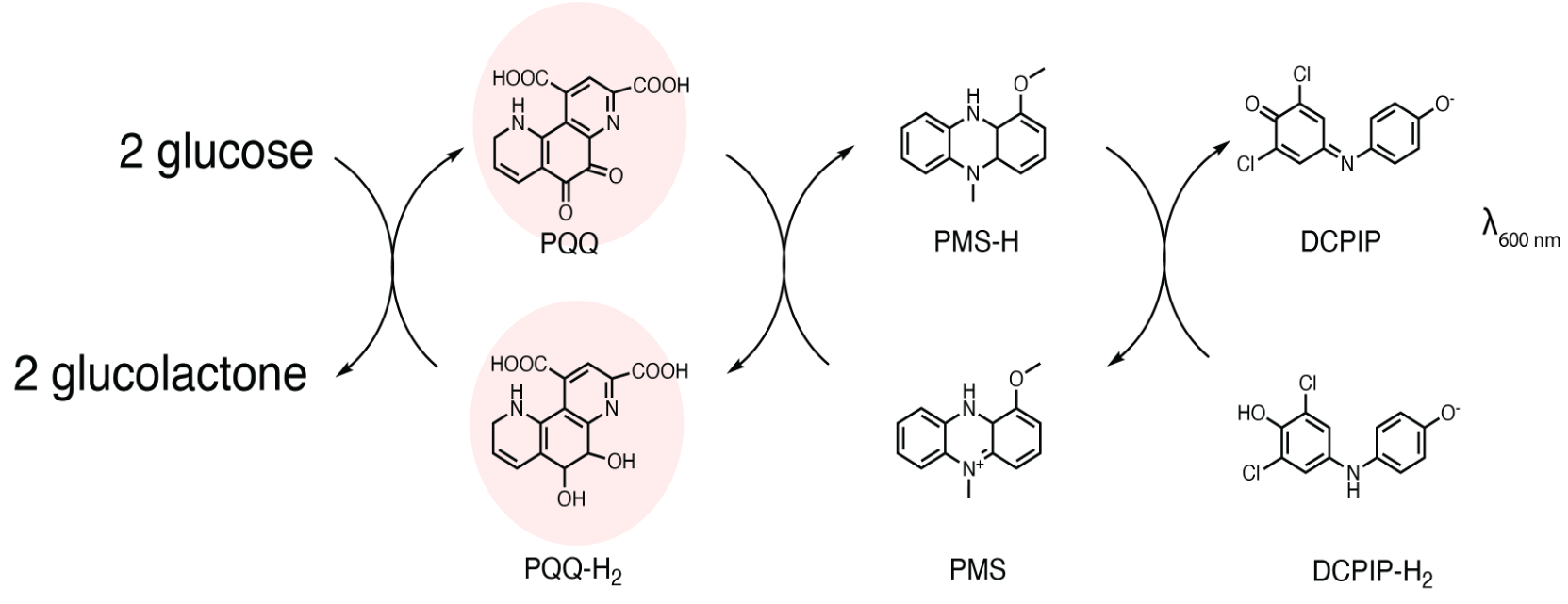


**Figure S3**. **GDH activity assay reaction.**


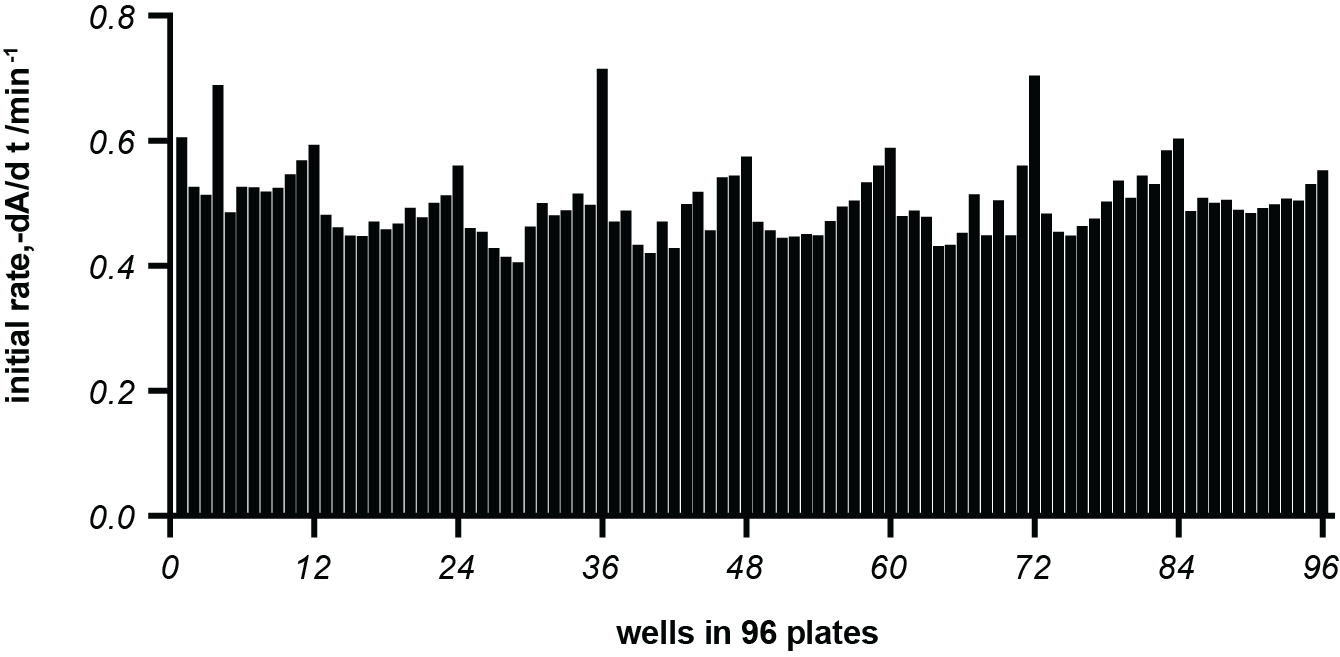


**Figure S4**. **Evaluating the screen method.** The GDH activity in the whole cell expressing wild-type GDH is plotted versus their well location in 96 plates.

**1.2.4 LBD-GDH variants screen**. As shown in Fig. S5, we first screened the LBD-GDH library for the variants capable of oxidizing glucose with 4-HT. In the colorimetric assay, the LBD-GDH active variants could turn the dark blue reagent solution yellow or colorless. We performed the screen in a Costar 96 flat transparent plate with 10 μL LBD-GDH cells mixed with a 180 μL reagent (section 2.2.3). Positive controls were prepared with cell expression wide-type GDH. Negative control was made with 10 μL cells harboring empty vectors to eliminate the interference of reagent decay over time. By arbitrarily comparing the color decay to the control group, LBD-GDH variants showing GDH activity within 5 minutes were pursued to the second screen round.

In the second round, 10 μL active LBD-GDH cells were mixed with 180 μL reagent and 10 μL DMSO or 4-HT (1mM dissolved in DMSO). Absorption at 600 nm was recorded every 15 s for 10 min by Tecan Spark plate reader at room temperature with orbital shaking at 500 rpm. Biologically independent experiments (n = 3) were performed in this screen round. The data were collected and processed with two-tailed, independent t–tests for P value. Only these variants showed statistically different (P < 0.05) activities with 4-HT and DMSO were identified as allosteric variants. Their change degree ($\frac{\Delta v}{v=\frac{\left| v_{ligand}-v_{dmso} \right|}{v_{dmso}}}$) is shown in Fig. S6.


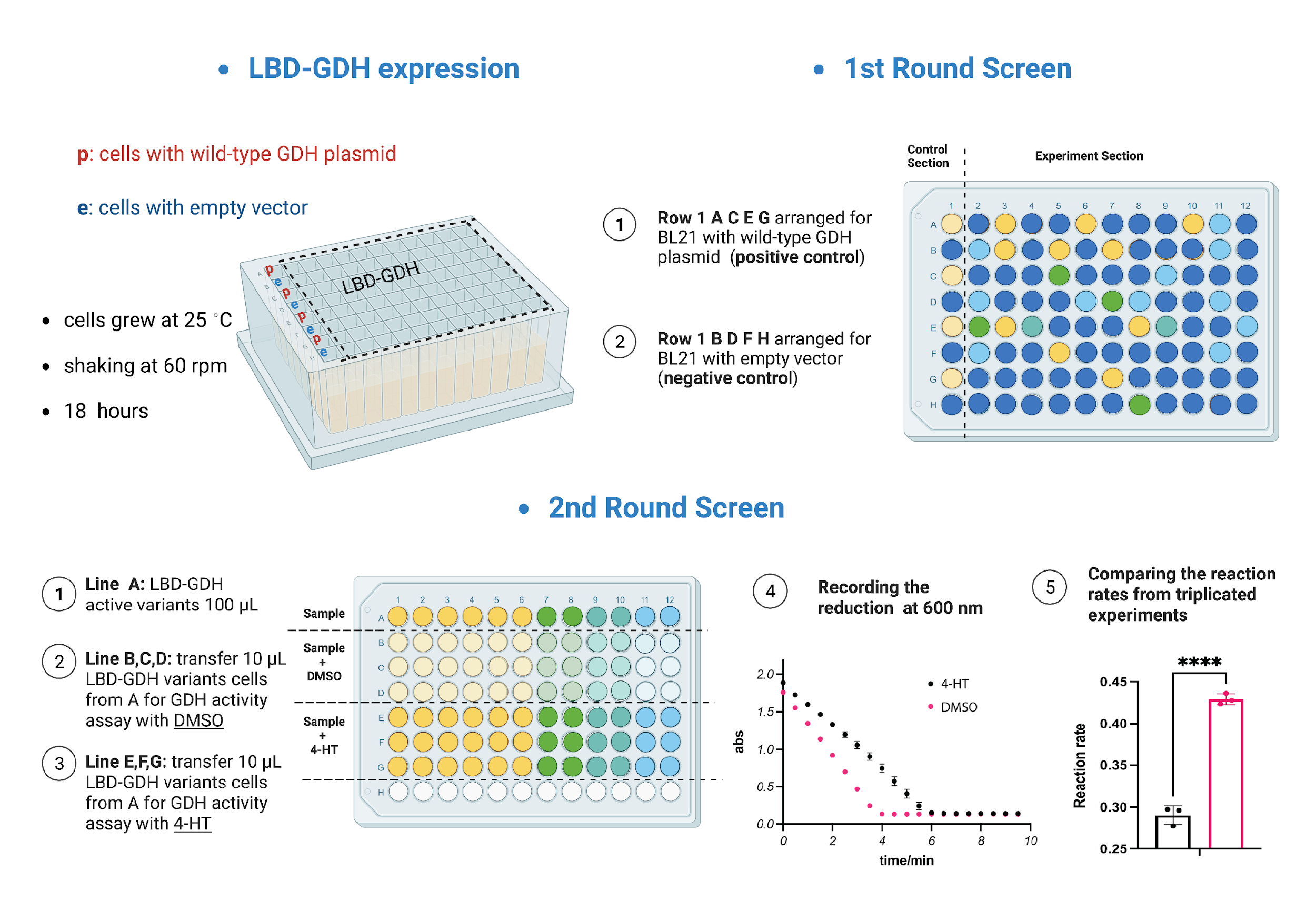


**Figure S5**. **Screen workflow, plate layout and sample data.** Created with BioRender.com

**
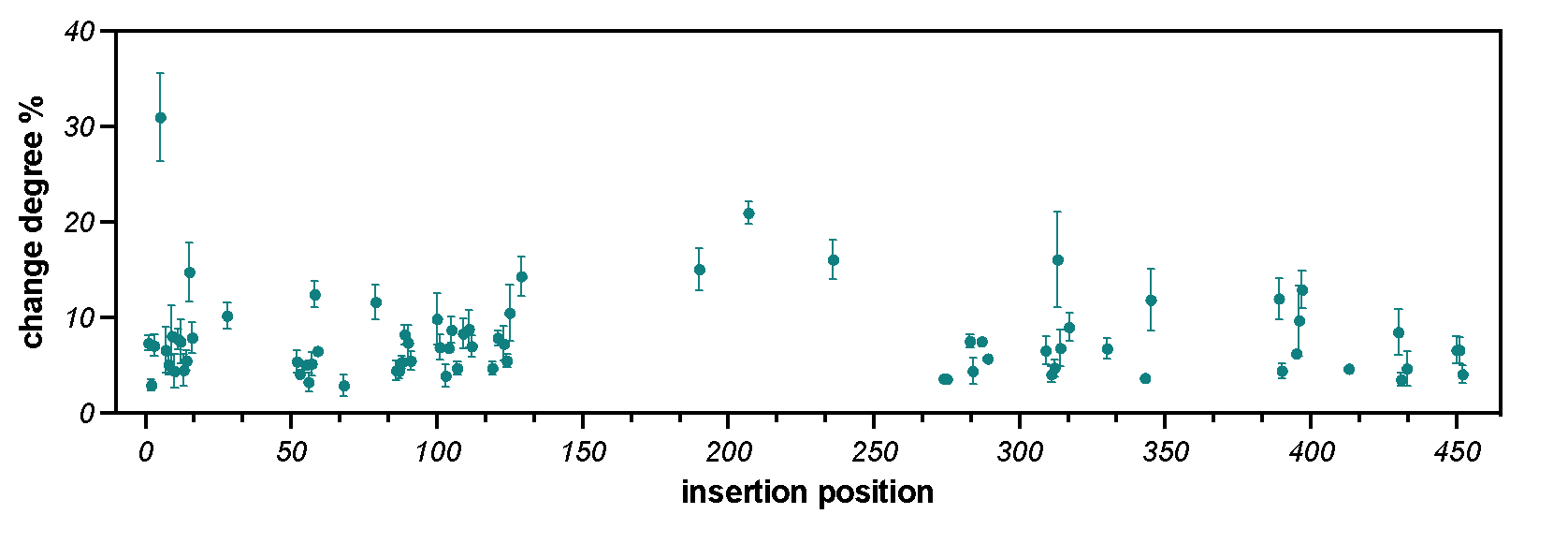
**

**Figure S6**. **Profile of allosteric values (change degree) for LBD inserted GDH variants.** Values are shown in mean ± sem with independent biological experiments (n ≥ 3).

**1.3** **The rationale for designing GDH-5E^+^**

Our screen unveiled specific locations for LBD insertion within GDH that enabled the resulting protein to be regulated by 4-HT. Since these locations were located within flexible regions in GDH (loops), these data suggested that conformational flexibility was important to allow allosteric changes in structure upon 4-HT binding (refer to Figure 2C). Building upon this discovery, our objective was to amplify the allosteric effect by augmenting flexibility by incorporating a flexible linker at previously identified allosteric positions identified through screening. We assumed the existence of an allosteric propagation pathway that traverses via Thr 5 and PQQ. We extended this potential allosteric structural rearrangement and incorporated residues 89, 207, and 104 into it (see Figure S7a).

In selecting the most suitable site, we hypothesized that two criteria would facilitate the propagation of conformational signals within the protein: (1) the even spacing of components and (2) the presence of most allosteric sites at the splitting interface. We depicted the theoretically completely split configurations of GDH-5E at positions 104, 89, or 207 (refer to Figure S7b-d). As these structures illustrate, splitting at positions 5 and 89 does not yield evenly distributed components. In contrast, compared to site 204, breaking at site 104 exposes more allosteric sites, particularly on the back of the N-terminus. Consequently, we opted to insert the flexible linker at Lys104.

**^
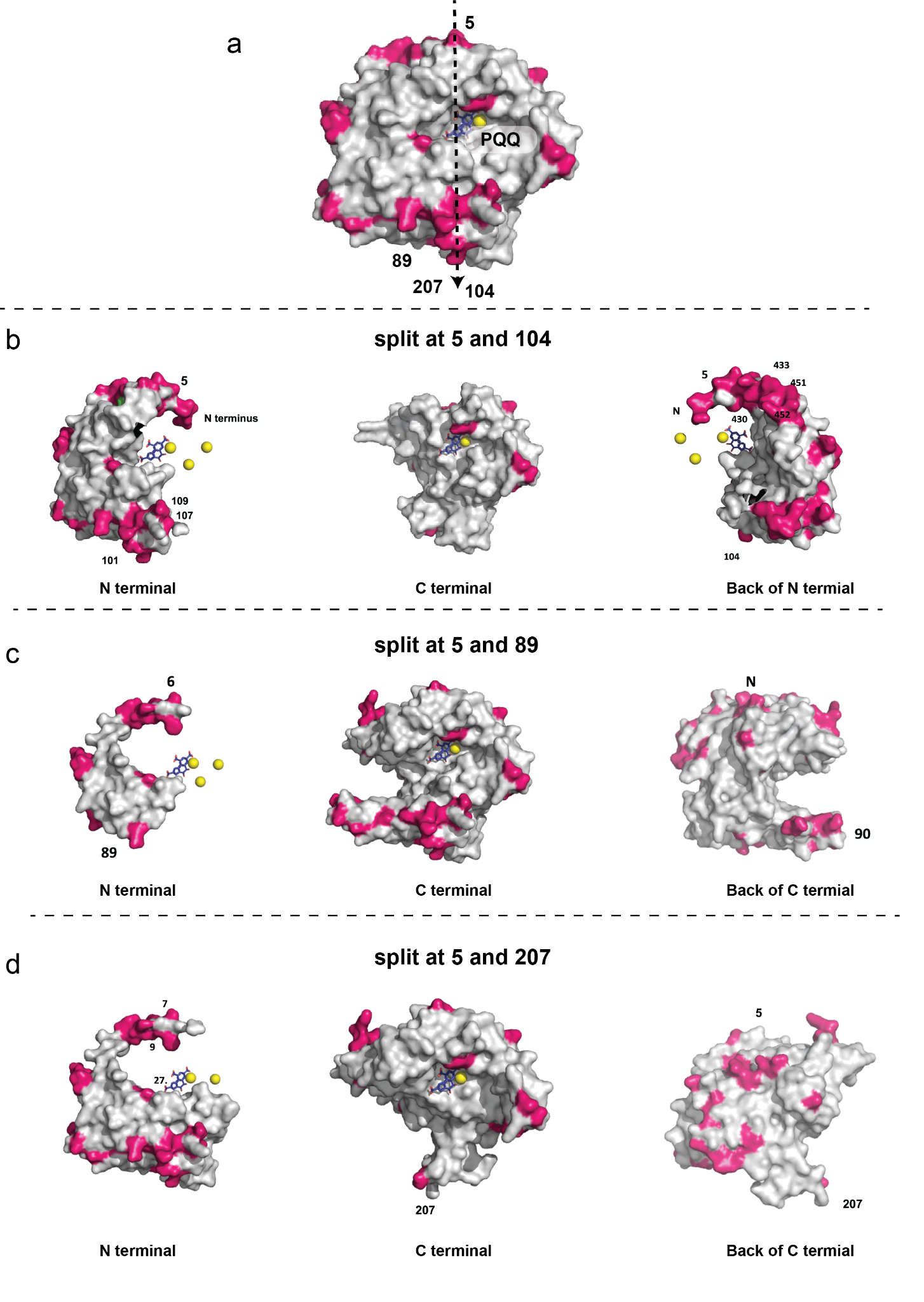
^**

Figure S7. **The rationale for designing GDH-5E+.** (a) Hypothetical allosteric propagation pathway in PQQ-GDH. (b-d) Hypothetical split structures of GDH-5E at 104, 89 or 207.

**2. Characterization of allosteric LBD-GDH**

**2.1. LBD-GDH expression and purification**

**2.1.1 Protein amino acids (AA) sequence.** Wild type-GDH, GDH-5E and GDH-5E^+^ were shown here. The black letters represent the GDH AA sequence, the green letters represent the LBD AA sequence, and the pink letters represent linkers.

**Wild-type GDH** HHHHHHDVPLTPSQFAKAKSENFDKKVILSNLNKPHALLWGPDNQIWLTERATGKILRVNPESGSVKTVFQVPEIVNDADGQNGLLGFAFHPDFKNNPYIYISGTFKNPKSTDKELPNQTIIRRYTYNKSTDTLEKPVDLLAGLPSSKDHQSGRLVIGPDQKIYYTIGDQGRNQLAYLFL

PNQAQHTPTQQELNGKDYHTYMGKVLRLNLDGSIPKDNPSFNGVVSHIYTLGHRNPQGLAFTPNGKLLQSEQGPNSDDEINLIVKGGNYGWPNVAGYKDDSGYAYANYSAAANKSIKDLAQNGVKVAAGVPVTKESEWTGKNFVPPLKTLYTVQDTYNYNDPTCGEMTYICWPTVAPSSAYVYKGGKKAITGWENTLLVPSLKRGVIFRIKLDPTYSTTYDDAVPMFKSNNRYRDVIASPDGNVLYVLTDTAGNVQKDDGSVTNTLENPGSLIKFTYKAK

**GDH-5E**

HHHHHHDVPLTSGKKNSLALSLTADQMVSALLDAEPPILYSEYDPTRPFSEASMMGLLTNLADRELVHMINWAKRVPGFVDLTLHDQVHLLECAWLEILMIGLVWRSMEHPGKLLFAPNLLLDRNQGKCVEGMVEIFDMLLATSSRFRMMNLQGEEFVCLKSIILLNSGVYTFLSSTLKSLEEKDHIHRVLDKITDTLIHLMAKAGLTLQQQHQRLAQLLLILSHIRHMSNKGMEHLYSMKCKNVVPLYDLLLEMLDAHRLHAPLSPSQFAKAKSENFDKKVILSNLNKPHALLWGPDNQIWLTERATGKILRVNPESGSVKTVFQVPEIVNDADGQNGLLGFAFHPDFKNNPYIYISGTFKNPKSTDKELPNQTIIRRYTYNKSTDTLEKPVDLLAGLPSSKDHQSGRLVIGPDQKIYYTIGDQGRNQLAYLFLPNQAQHTPTQQELNGKDYHTYMGKVLRLNLDGSIPKDNPSFNGVVSHIYTLGHRNPQGLAFTPNGKLLQSEQGPNSDDEINLIVKGGNYGWPNVAGYKDDSGYAYANYSAAANKSIKDLAQNGVKVAAGVPVTKESEWTGKNFVPPLKTLYTVQDTYNYNDPTCGEMTYICWPTVAPSSAYVYKGGKKAITGWENTLLVPSLKRGVIFRIKLDPTYSTTYDDAVPMFKSNNRYRDVIASPDGNVLYVLTDTAGNVQKDDGSVTNTLENPGSLIKFTYKAK

**GDH-5E^+^**

HHHHHHDVPLTSGKKNSLALSLTADQMVSALLDAEPPILYSEYDPTRPFSEASMMGLLTNLADRELVHMINWAKRVPGFVDLTLHDQVHLLECAWLEILMIGLVWRSMEHPGKLLFAPNLLLDRNQGKCVEGMVEIFDMLLATSSRFRMMNLQGEEFVCLKSIILLNSGVYTFLSSTLKSLEEKDHIHRVLDKITDTLIHLMAKAGLTLQQQHQRLAQLLLILSHIRHMSNKGMEHLYSMKCKNVVPLYDLLLEMLDAHRLHAPLSPSQFAKAKSENFDKKVILSNLNKPHALLWGPDNQIWLTERATGKILRVNPESGSVKTVFQVPEIVNDADGQNGLLGFAFHPDFKNNPYIYISGTFKNPKSGRPGSLSSTDKELPNQTIIRRYTYNKSTDTLEKPVDLLAGLPSSKDHQSGRLVIGPDQKIYYTIGDQGRNQLAYLFLPNQAQHTPTQQELNGKDYHTYMGKVLRL

NLDGSIPKDNPSFNGVVSHIYTLGHRNPQGLAFTPNGKLLQSEQGPNSDDEINLIVKGGNYGWPNVAGYKDDSGYAYANYSAAANKSIKDLAQNGVKVAAGVPVTKESEWTGKNFVPPLKTLYTVQDTYNYNDPTCGEMTYICWPTVAPSSAYVYKGGKKAITGWENTLLVPSLKRGVIFRIKLDPTYSTTYDDAVPMFKSNNRYRDVIASPDGNVLYVLTDTAGNVQKDDGSVTNTLENPGSLIKFTYKAK

**2.1.2 Protein expression.** *E. coli* BL 21 harboring GDH, GDH-5E, or GDH-5E^+^ plasmids were cultured in 500 mL LB broth with 50 µg/mL kanamycin. Cultures were grown in a 2 L flask at 37 ^◦^C, shaking at 250 rpm, until OD_600_ reached ~ 0.8. Protein expression was triggered by adding IPTG (50 µM for GDH; 200 µM for GDH-5E and GDH-5E^+^). GDH was expressed at 25 ^◦^C for 4 h with shaking at 180 rpm, while GDH-5E, and GDH-5E^+^ were expressed at 15 ^◦^C for 15 hours with shaking at 120 rpm. Cells were harvested by centrifugation (8000 x g, 5 minutes) and stored at –80 ^◦^C.

**2.1.3 Protein purification and reconstitution.** Frozen cells (~ 30 g) were thawed and resuspended in 150 mL buffer (50 mM HEPES, 10 mM imidazole, 300 mM NaCl, 3 mM CaCl_2_ and 5 mM beta-mercaptoethanol, pH 7) with lysozyme and DNase, and lysed with a AVESTIN EmulsiFlex-C3 homogenizer. The lysate was clarified at 22,000 x g for one hour to precipitate the cell debris. Protein is bound on a 5 mL Histrap FP Ni-NTA column (Cytiva) on an FPLC and washed with a 30 % gradient of imidazole buffer (50 mM HEPES, 300 mM imidazole, 300 mM NaCl, 3 mM CaCl_2_ and 5 mM beta-mercaptoethanol, pH 7). The protein was eluted in 40 % imidazole buffer

and loaded on a 15 mL HiTrap Desalting column (Cytiva) to remove imidazole. PQQ was added to the protein solution at a 2:1 molar ratio. The mixture was stirred for 30 min. Excess PQQ was removed by loading again on the desalting column. The purity of the protein was confirmed by SDS–PAGE (Fig. S8), and the concentration was measured with Bradford assay.


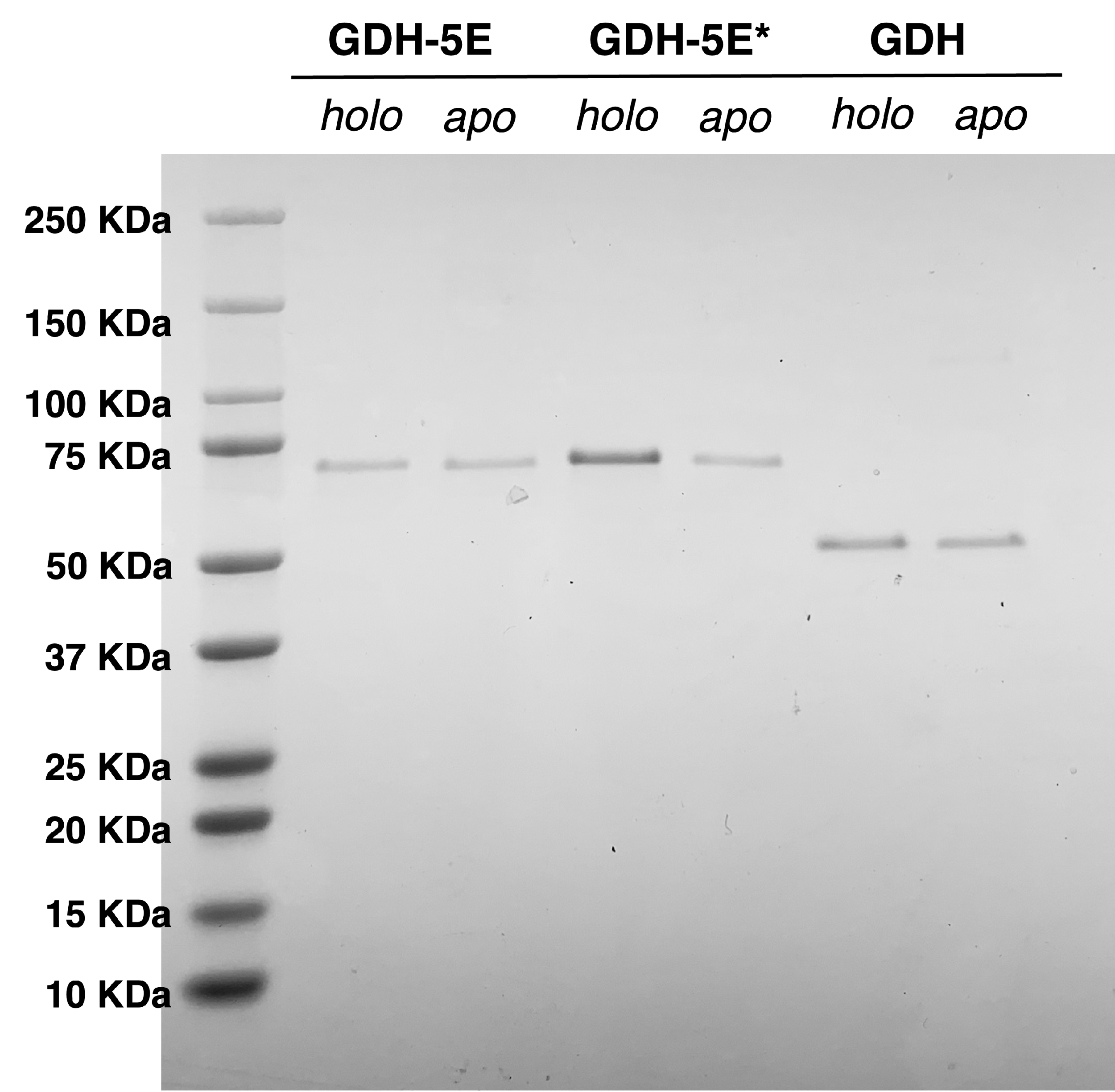


**Figure S8.** **SDS**–**PAGE analysis of proteins.** Each line contains ~1 μg protein sample.

**2.2. LBD-GDH characterization**

**2.2.1 UV**–**vis.** Protein absorption spectra were taken in 50 mM potassium phosphate buffer, pH 7.0, with 10 nmol/mL protein. Compared with holo GDH, the PQQ absorption peak blue-shifted to 330 nm in holo GDH-5E (Fig. S9).

**
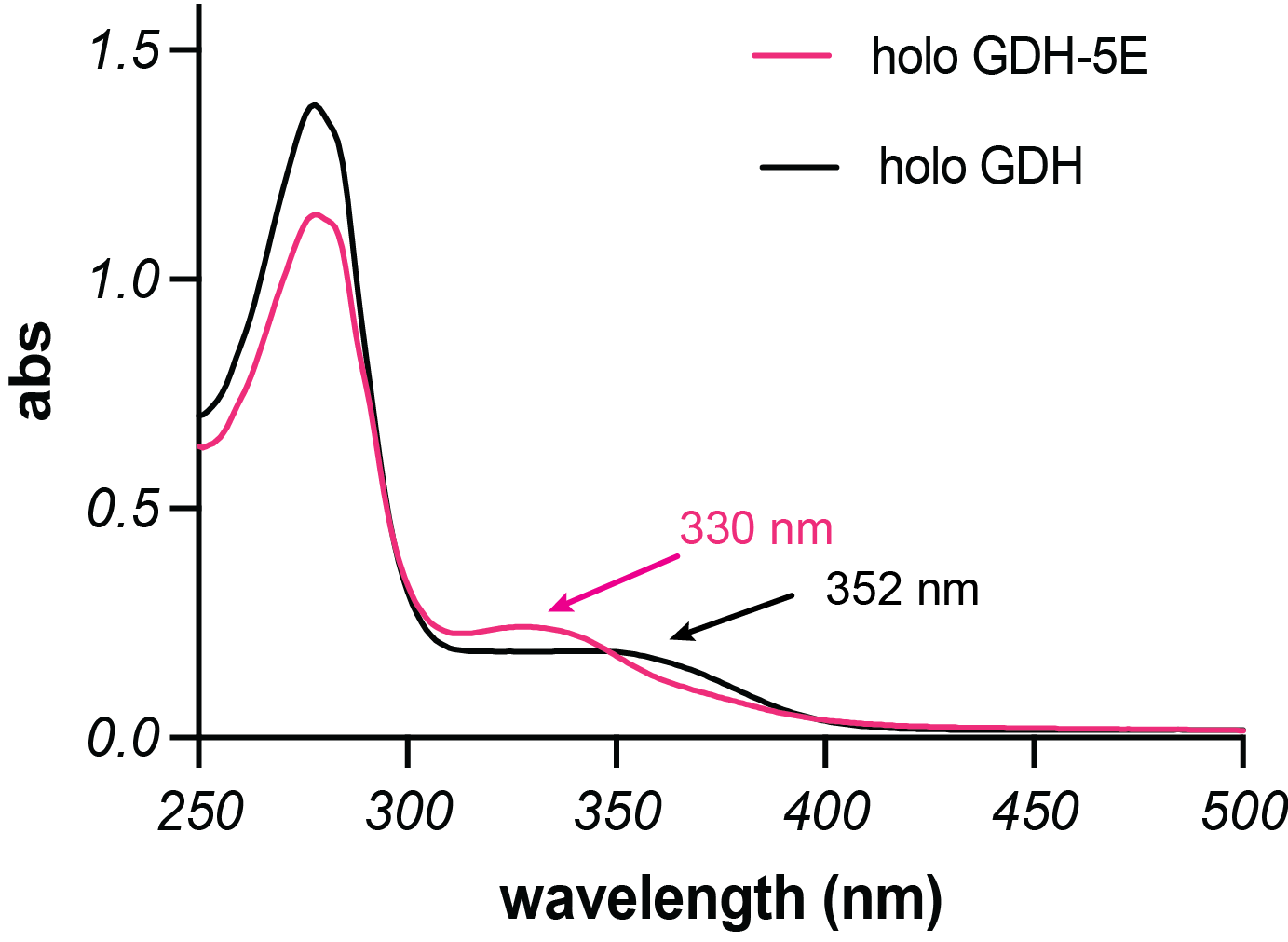
**

**Figure S9**. **Absorption spectra of GDH and GDH-5E.** 10 nmol protein was dissoved in 1 mL 50 mM potassium phosphate buffer, pH 7.0 in cuvette.

After adding 5 mM glucose, this peak barely shifts (Fig.S10). In contrast, glucose reduction caused a sharp peak at 338 nm, which aligned well with the previous report (*4*). These suggested that GDH-5E were in its reduced form. Adding 4-HT (1 µM) didn’t shift the PQQ peak, which indicated that the 4-HT does not affect the redox potential of GDH-5E.


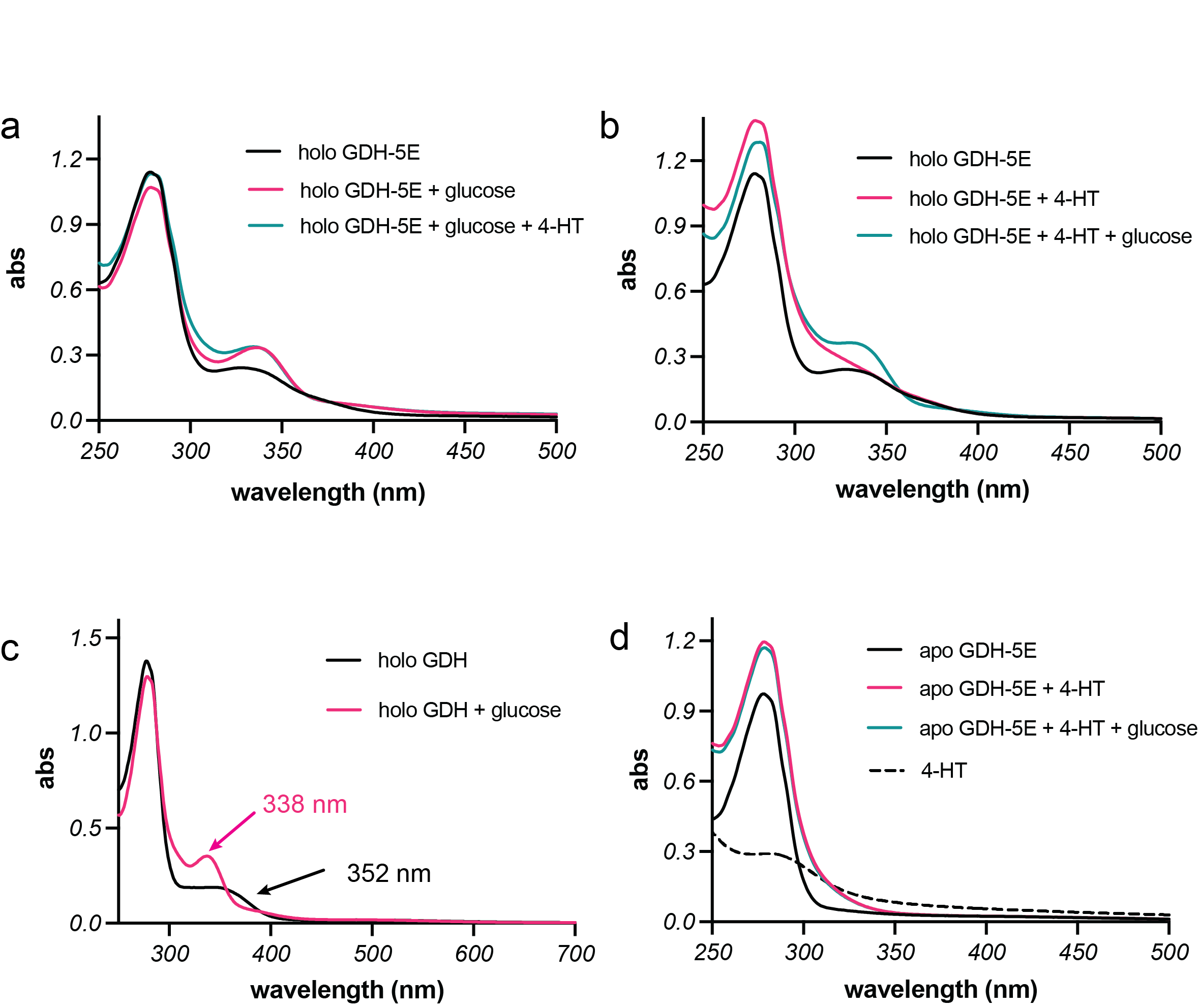


**Figure S10. Absorption spectra of GDH-5E with glucose and 4-HT.** 10 nmol protein was dissolved in 1 mL 50 mM potassium phosphate buffer, pH 7.0, in a cuvette. (a) GDH-5E sequentially added glucose, then 4-HT. (b) GDH-5E sequentially added 4-HT, then glucose. Control experiments were prepared with GDH (c) and apo GDH-5E (d).

**2.2.2 Protein activity.** A reagent solution containing 47 mL MOPS buffer (10 mM, pH 7), 1 mL DCPIP (20 mg dissolved in 5 mL of H_2_O), 1 mL PMS (45 mg in 5 mL of H_2_O, freshly prepared and kept in the dark) were prepared for each use. The assay was performed on a flat transparent 96 plate in triplicates. Since GDH has a high turnover rate, each test requires only 5-10 ng protein. The experiments were prepared by mixing 10 µL protein with a 180 μl reagent solution. Reactions were initialized by adding 10 µL glucose and recorded every 15s for 10 min by Tecan Spark plate reader with orbital shaking at 500 rpm. The coefficient of variance is 7.6 %.

We characterized the proteins’ activities with various glucose concentrations, ranging from 0 mM to 80 mM, and fitted them into the Michaelis–Menten kinetics (MMK) curves (Fig. S11). The fitted K_m_ and V_m_ were summarized in Table S2.

**Figure S11. MMK curves with GDH-5E^+^, GDH-5E and GDH.** Values are shown in mean ± sd with independent experiments (n = 3).

**Table S2. Summary of MMK**


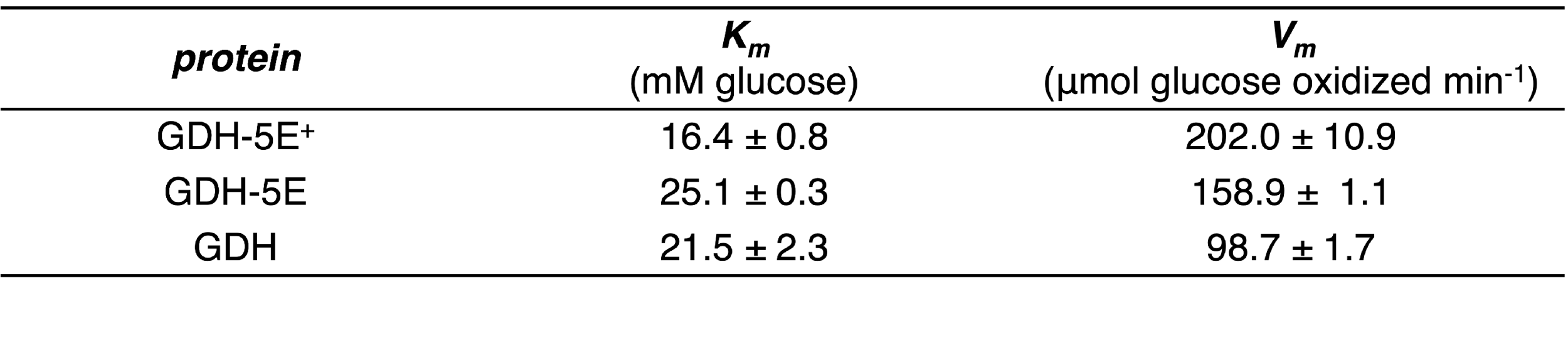


**2.2.3 Allosteric effect.** According to LBD crystal structure, LBD’s ligand-dependent conformational change is mainly mediated by helix 12 (orange colored). When LBD harbors ES (17β-estradiol), the N-C termini distance is ~33 Å. While 4-HT binding moves the helix 12 away from the binding pocket, resulting in a shorter termini distance of ~23 Å (Fig. S12).


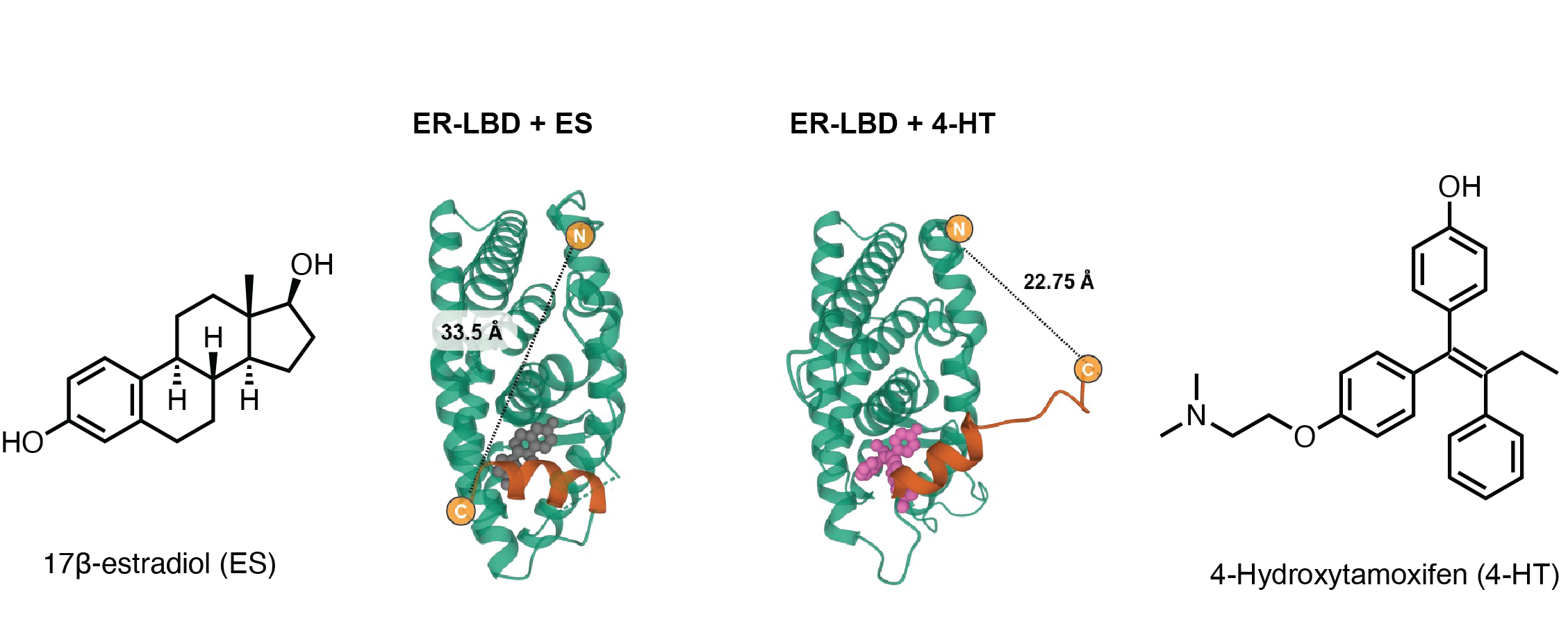


**Figure S12**. **Crystal structure of LBD with ES or 4-HT.** ER-LBD with ES (PDB: 1ERE); ER-LBD with 4-HT (PDB: 3ERT). The chemical structure of ES and 4-HT were also shown.


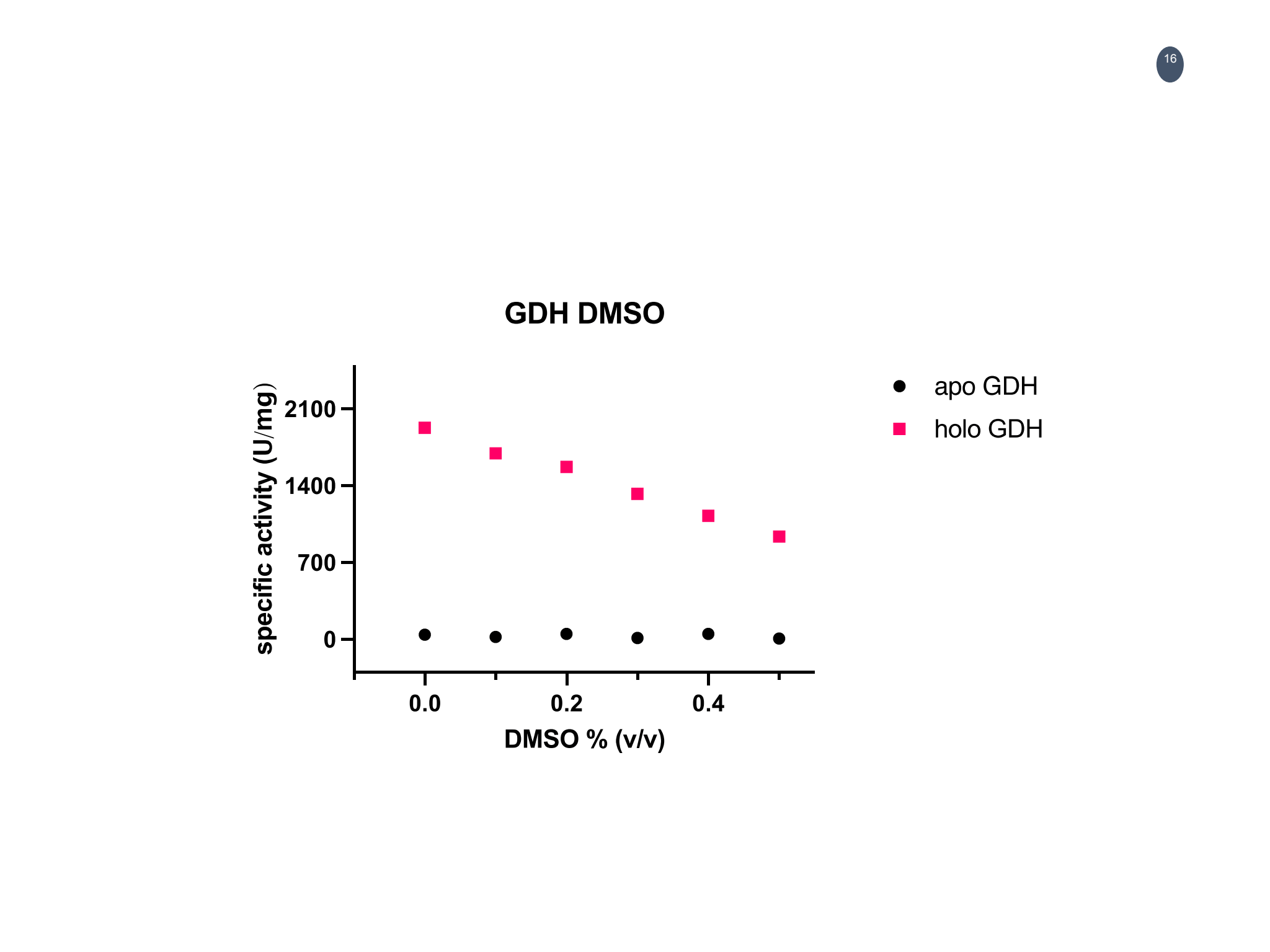


**Figure S13**. **The inhibition of DMSO on GDH.** The reactions were performed with 10 ng GDH in 200 μL solution with DCPIP, PMS and glucose in 10 mM MOPS, pH 7. Values are shown with independent experiments (n = 1).

**2.2.4 Protein stability.** The protein stability was evaluated by comparing the remaining activity after being stored at – 80 ^◦^C for three months. The protein was thawed on ice and tested for the activities described in section 3.2.2.

**Figure S14**. **The remaining activity of GDH-LBD.** Values are shown in mean ± sd with independent experiments (n = 3).

**3. Creating a 4-HT amperometric sensor with GDH-5E^+^**

**3.1 GDH-5E^+^ electrode preparation**

**3.1.1 Fc-LPEI synthesis**. Fc**–**LPEI was synthesized as previous reported.(*5, 6*) High molecular weight polyethylenimine (LPEI, 0.100 g, 2.33 mmol) was dissolved in a mixture of acetonitrile (7 mL) and methanol (3 mL) at 80 °C. A solution of 3-bromopropyl-dimethylferrocene (0.137 g, 0.45 mmol) in acetonitrile (2 mL) was added to the stirring LPEI solution at 80 °C, and the mixture was stirred at 90-100 °C for 24 hours. The reaction mixture was cooled to room temperature and the solvent was removed under reduced pressure. Excess ferrocene was removed by soaking the resulting polymer in diethyl ether (10 mL) for 1 hour at room temperature. The diethyl ether was decanted and excess solvent was removed under reduced pressure. The final product was a brown malleable solid (0.169 g, 71% yield). ^1^H-NMR in CD_3_OD: δ (ppm) 1.67 (2H, C-CH_2_-C), 1.94 (6H, Fc(-CH_3_)), 2.30 (2H, Fc-CH_2_-C), 2.50 - 3.10 (16H, N-CH_2_-C, polymer backbone), 3.87 (7H, Fc-H).

**3.1.2 AvCarb carbon electrode preparation**. Nonwet-proofed AvCarb carbon paper electrodes were cut into strips (3 cm x 0.5 cm). Except for the designed electrode surface area, the rest of the strip was dipped into melting paraffin wax to seal the conductive surface. Before electrochemical

testing, the exposed end was coated with protein/Fc-LPEI hydrogel for analysis, while the waxed end was used as an electrochemical connection point.

**3.1.3 GDH-5E^+^/Fc**–**LPEI hydrogel film preparation.** 6 μL GDH-5E^+^ (16 mg/mL) was added to 14 μl of Fc–LPEI solution (12 mg/mL in H_2_O). The mix was vortexed before adding EGDGE (0.75 μL, 4.4 % by volume in H_2_O). The resulted mixture (~20 μL) was then drop-coated onto a glassy carbon electrode (diameter, 3 mm) in 3 μL aliquots or AvCarb carbon electrode (0.5 cm x 0.5 cm) in 10 μL aliquots. The resulted electrodes were cross-linked at 4 ^◦^C for 6 h.

**3.2 Cyclic voltammetry (CV).**

CVs were performed with three electrodes: AvCarb coated with GDH-5E^+^/Fc–LPEI as the working electrode, a saturated calomel electrode (SCE) as the reference, and a platinum mesh counter electrode. Experiments were performed at room temperature in 5 mL MOPS buffer (100 mM, pH 7.0). All the protein hydrogel coated electrodes were electrochemically conditionedwith 10 cycles of scanning at 100 mV/s. The electrochemical activities of GDH-5E^+^ were measured by CV scan at 20 mV/s in a stationary solution for three cycles. The third cycle was used for analysis and reporting. Reported potentials were referenced to the standard hydrogen electrode (SHE) by adding 244 mV to the measured values.

**3.3 Amperometric i–t curves.**

A fixed potential of 300 mV vs SCE was first applied to working electrodes for 100 s. This step could effectively decrease the double**–**layer charging current in the following tests. Electrodes were soaked in a stirred solution of 100 mM MOPS, pH 7. Glucose was injected into the electrolytes after the current stabilized (usually 180 seconds).

The proposed working model was that LBD undergoes a conformational change following 4-HT binding. The resulted structure had a lower glucose oxidation capacity. To validate this hypothesis, we first added 10 nM 4-HT into electrolytes. Electrodes were soaked in electrolytes for 3 minutes before sequentially spiked glucose (Fig. S15a). Compared to the electrodes with ES, the electrodes in the 4–HT–containing system showed ~ 22 % decreased current at each glucose spiking. Although we observed the current difference at initial injections, significant differences were achieved when glucose ≥150 μM. Further increasing the 4-HT concentration to 12.5 μM, we observed similar electrochemical current repression from 4-HT. This result aligned with the previous assay results in Figure 3B that the GDH-5E^+^ lacks dynamic range for 4-HT concentration.

**
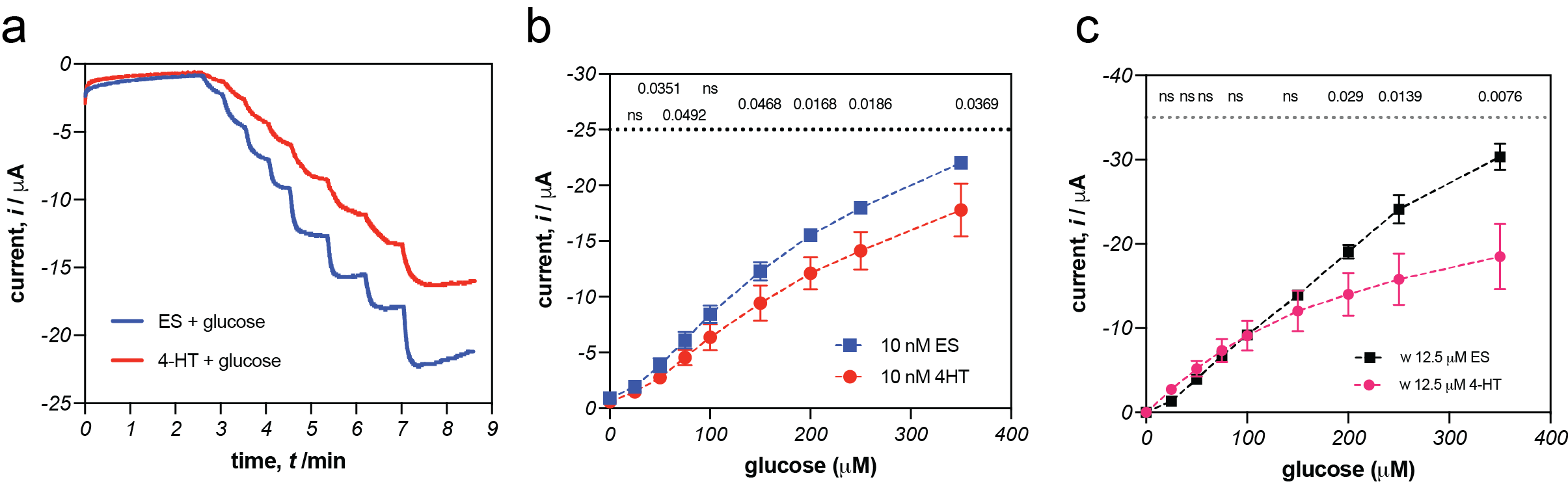
**

**Figure S15. Amperometric experiment with GDH-5E^+^ electrodes pre-soaked with 4-HT or ES**. (a) Representative amperometric i-t traces. Glucose solution was added successively to 100 mM MOPS electrolyte (pH = 7) with 10 nM 4-HT or ES. Experiments were performed under an applied potential of 544 mV vs. SHE with AvCarb electrodes (0.25 cm^2^) and stirring. (b) Summary and statistical analysis of triplicated amperometric experiments with electrolytes containing 10 nM ES or 4-HT. (c) Summary and statistical analysis of triplicated amperometric experiments with electrolytes containing 12.5 μM ES or 4-HT. Values are shown in mean ± sd with independent biological experiments (n = 3). Two-tailed t-test analysis.

**3.4 Electrochemistry with human blood sample.**

Single donor human whole blood using K2 EDTA as the anticoagulant was purchased from Innovative Research. (Caution: the received blood sample has had the FDA-required viral testing and used following universal precautions.)

CVs were performed with AvCarb electrodes coated with GDH-5E^+^/Fc–LPEI. After adding 200 μL whole blood to 5 mL MOPS buffer (100 mM, pH 7.0) under stationary condition, we observed oxidative current appearing on GDH-5E^+^ electrodes (Fig. S16a). This oxidative response was less apparent than on the glassy carbon electrodes because of the high capacity natural of the AvCarb. The blank control electrodes were prepared by immobilizing bovine serum albumin (BSA, 20 mg/mL) with Fc–LPEI. The current decreased after adding blood (Fig. S16b), which might result from the conductivity decline caused by blood cells precipitating on the electrode.

In amperometry, we stirred the solution vigorously (1100 rpm) to mix blood samples into electrolytes quickly. At the applied potential of 300 mV vs. SCE, the GDH-5E^+^ showed a stepwise current increase after each spiking of blood (Fig. S16c), while the BSA electrode had no response to the blood (Fig. S16d). These controls claimed that although whole human blood contains complex species, our electrochemical system is specific to glucose.


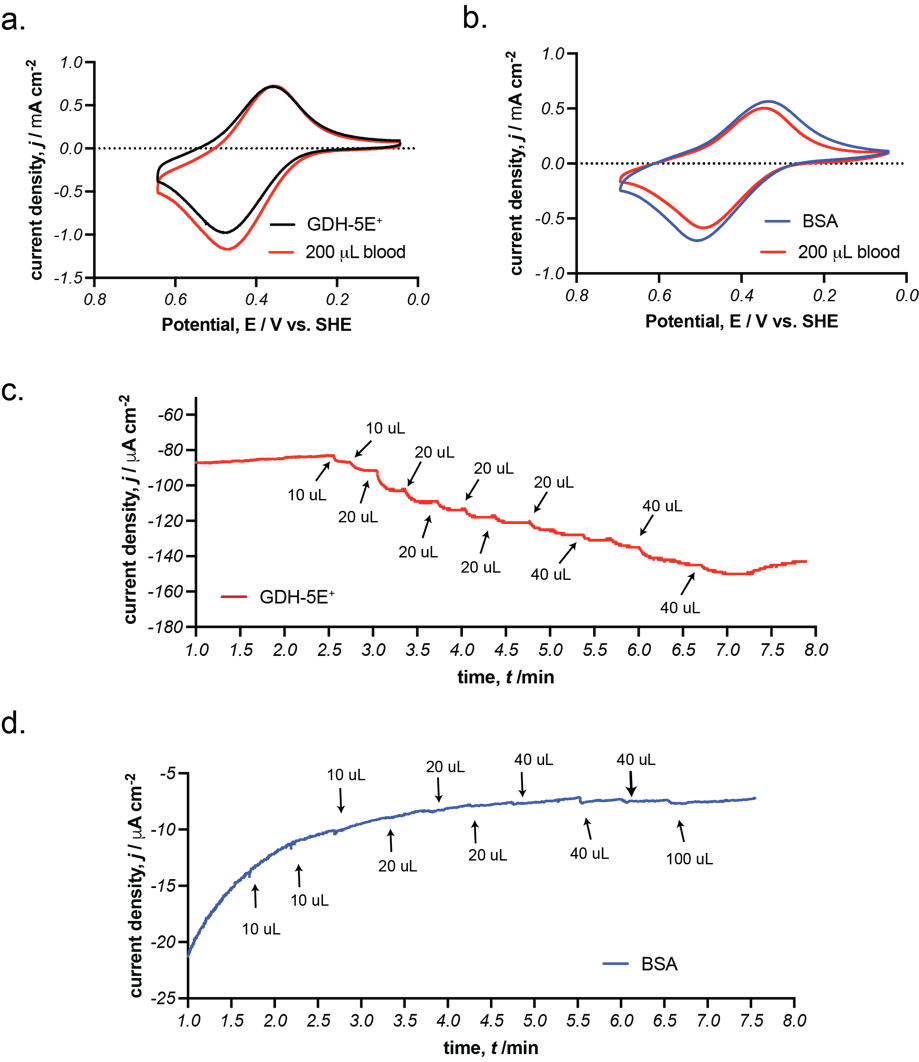


**Figure S16. Electrochemistry of GDH-5E^+^ with whole human blood.** CV of GDH-5E^+^ (a) and BSA (b) with a scan rate of 10 mV/s in a stationary solution. Amperometric i–t of GDH-5E^+^ (c) and BSA (d) with an applied potential of 544 mV vs. SHE with stirring. 100 mM MOPS buffer.

**4. Creating a self-powered sensor**

**4.1 Laccase cathode**

**4.1.1 Laccase cathode preparation.** Laccase from *Trametes* versicolor (≥ 0.5 U/mg) was purchased from Sigma–Aldrich. The enzyme deposition solution was prepared by suspending laccase (30 mg) in 75 μl of 200 mM citrate–phosphate buffer (pH 4.5), followed by the addition of 7.5 mg of anthracene–modified multi-walled carbon nanotube (An-MWCNTs).(*5*) The mixture was subjected to successive vortex/sonication steps until the ink was homogenous. TBAB-Nafion (25 μL)(*8*) was added, and a few more vortex/sonication steps were undertaken to promote thorough mixing. 30 μL ink was spread onto the AvCarb electrodes (1.25 cm x 0.8 cm) and dried at 4 ^◦^C overnight.

**4.1.2 CV of laccase electrode.** CVs of laccase biocathodes were performed in 200 mM citrate–phosphate buffer (pH 4.5) at 10 mV/s, as shown in Fig. S17. These catalytic currents under O_2_ or air reflect the turnover of the copper centers in the laccase. (*9*) During O_2_ reduction, the electrode supplies electrons to cycle the copper redox states in the laccase. Anthracene served as a docking site for the copper redox center, enabling electrons to directly transfer between the electrode and the laccase, i.e., direct electron transfer (DET). (*5*) The responses under N_2_ lack catalytic signals for O_2_ reduction, showing only the background capacitive charging current with laccase redox center ~725 mV vs SHE.


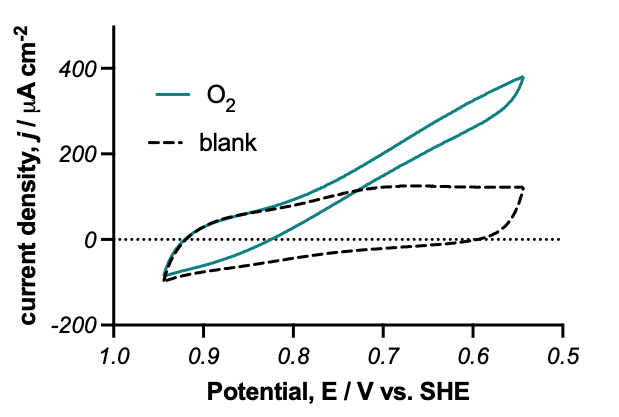


**Figure S17**. **Representative CV of laccase biocathodes.** Recorded in 200 mM citrate–phosphate buffer (pH 4.5) with 10 mV/s scan rate. Blank were performed under N_2_.

**4.2 Glucose/O_2_ enzymatic fuel cell (EFC).** EFCs were set up in a custom-made electrochemical cell, where a Nafion® 212 proton exchange membrane (PEM) was used to compartmentalize the anodic and cathodic chambers. In the anodic chamber, we use GDH-5E^+^/Fc–LPEI coated AvCarb (0.5 cm x 0.5 cm) as the anode, with 4 mL 100 mM MOPS as electrolytes. On the cathodic sides, we used laccase/An-MWCNTs/TBAB-Nafion coated AvCarb electrode (1.25 cm x 0.8 cm). This chamber contained 4 mL 200 mM citrate–phosphate buffer, pH 4.5, having O_2_ bubbled (Fig. S18).


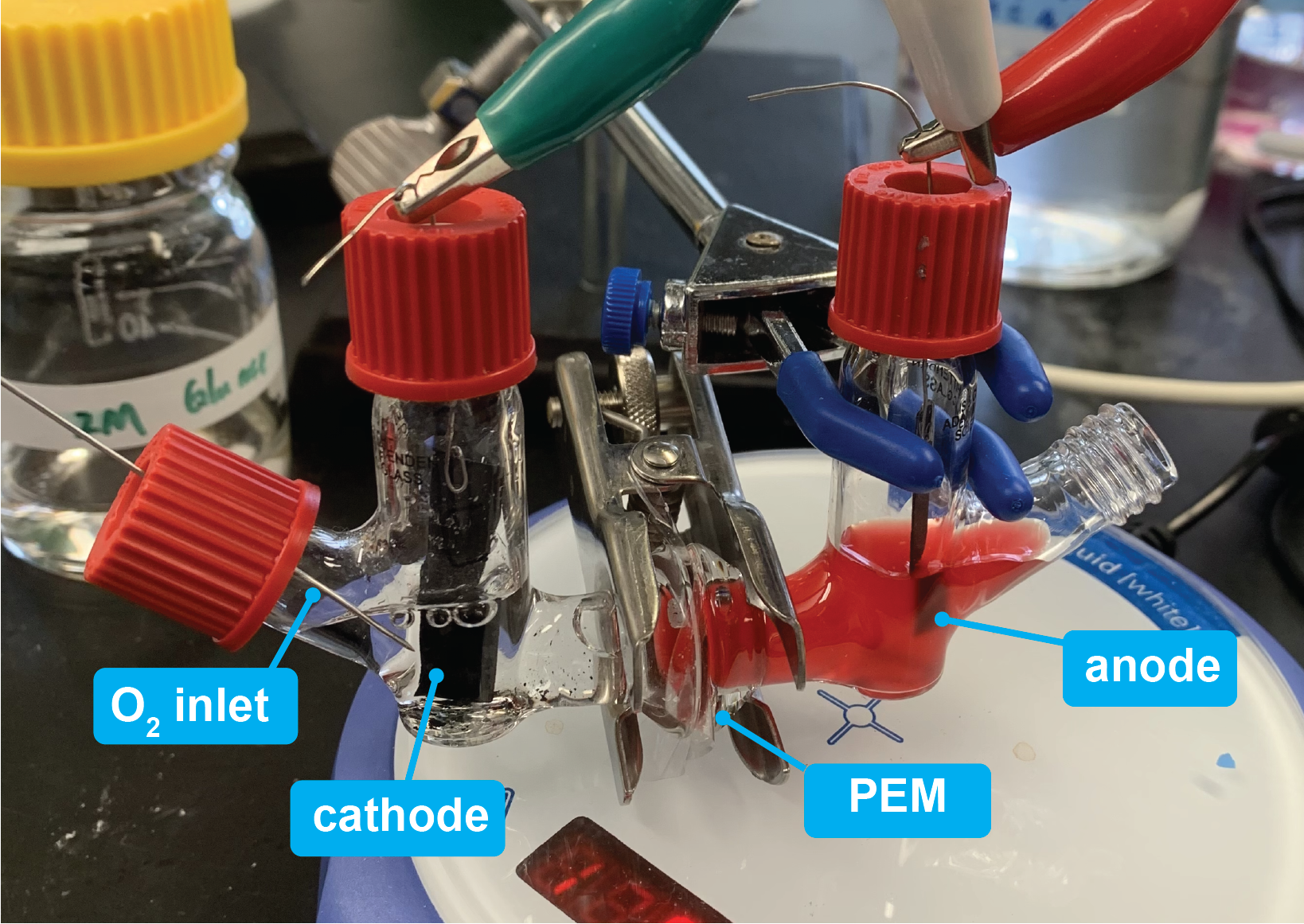


**Figure S18. A photo of the glucose/O_2_ EFC with blood sample in the anodic chamber.**

**4.3 A self-powered 4-HT sensor**

The capacity of EFC to sense 4-HT was explored by comparing the fuel cell parameters with or without 4-HT addition. We performed the linear polarization with 40 μL of 10 mM glucose comprising 1 % (v/v) 4-HT (10 μM, in DMSO). The resulting fuel cell showed J_max_ of 45 ± 2 μA /cm^2^ and P_max_ of 9.5 ± 0.5 μW/cm^2^, statistically lower than the EFC performing with ES (Fig. S19)


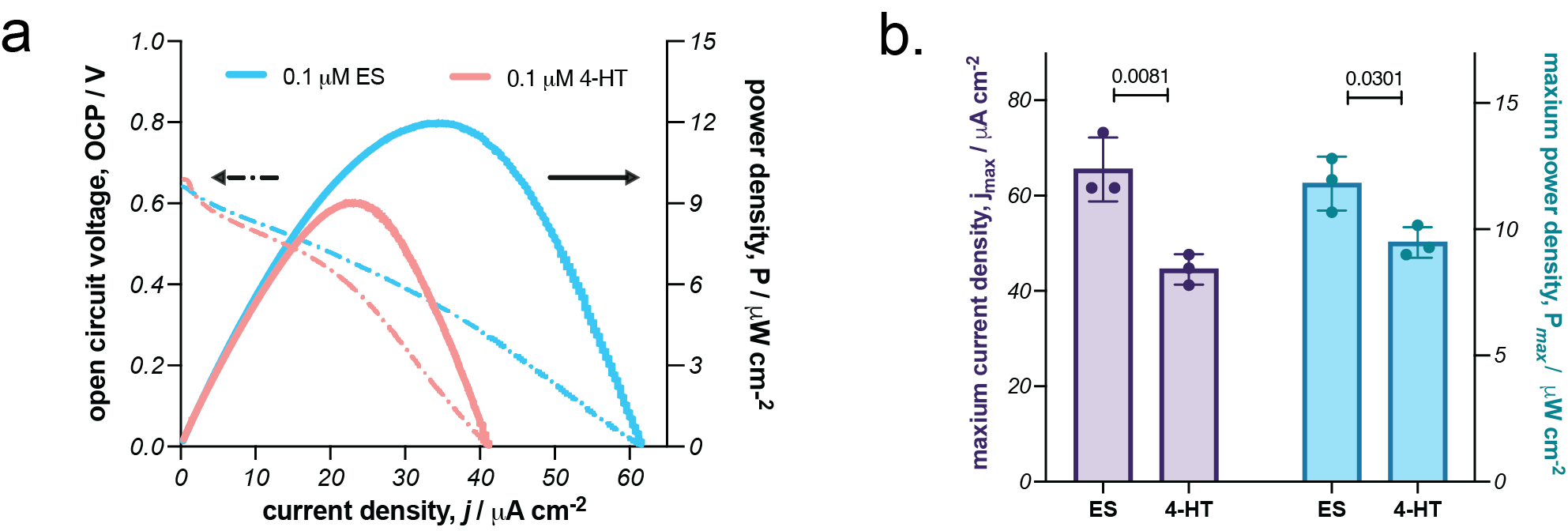


**Figure S19.** **A self-powered 4-HT sensor.** (a) Representative polarization and power curves of the glucose/O_2_ EFC with 0.1 mM glucose and 0.1 μM 4-HT or ES. Experiments were performed by linear sweep voltammetry (5 mV/s) from open circuit potential (OCP) until short circuit. (b) Summary and statistical analysis of triplicated polarization experiments. Data are collected from independent experiments (n=3) with two-tailed t-test analysis.

**Figure S20.** **Open circuit potential (OCP) of glucose/O_2_ EFC fueled with 200 μLblood containing 100 nM 4-HT or ES.** Values are shown in mean ± sd; two-tailed t-test (n =3).

**5. Coupling EFCs with OECT**

**5.1 OECT fabrication**.

**5.1.1 Fabricating OECT device.** The OECT devices were fabricated using a low-cost methodology. Glass slides were first cleaned with soap and DI water, then washed in isopropanol and acetone before blow-drying with clean, dry air. Later, thermal evaporation deposited Au (65 nm) on the cleaned glass slides, which were cut into glass strips (1.2 cm x 2.5 cm). Using a new single–edged razor blade, we cut a line along the glass strip, creating a channel with a length of 50 µm. By masking with Kapton tape, we defined a 1 mm width for the channel. Additional areas where Au was deposited were left uncovered to provide electrical contact for alligator clips. The slides were further subject to UV-Ozone treatment for 10 minutes to ensure effective adhesion to the OECT solution. An Ag/AgCl wire was prepared as the gate electrode.

**5.1.2 Preparing solution for OECT channel.** The channel of OECT was made of poly (3,4 ethylene dioxythiophene) doped with poly(styrene sulfonate) (PEDOT:PSS). An OECT solution was prepared by ultrasonicating PEDOT:PSS with ethylene glycol (5 % v/v) and (3-glycidyloxypropyl) trimethoxysilane (GOPS, 1 % w/w). 4-dodecylbenzenesulfonic acid (DBSA, 0.1 % v/v) was added to ensure uniform mixing. This OECT solution was spin-coated to the prepared channel at 1000 rpm for 45 seconds and accelerated at 200 rpm/s. The resulting devices were annealed at 1400 ^◦^C for 30 minutes.

**5.1.3 Transfer characteristic of OECT**. The OECT devices, under the common-source configuration, were tested using custom-made MATLAB software, which controls the source measure unites (SMU, Keithley 2612). Output and transfer curves of the devices were recorded before connecting the OECT to the EFC. Transfer characteristics (Fig. S21) of the OECT were measured in PBS electrolyte until three subsequent transfer curve sweeps had less than 1 % deviation compared to the preceding transfer curve. Upon reaching stable operation, the gate electrode was connected to the cathode of EFC.


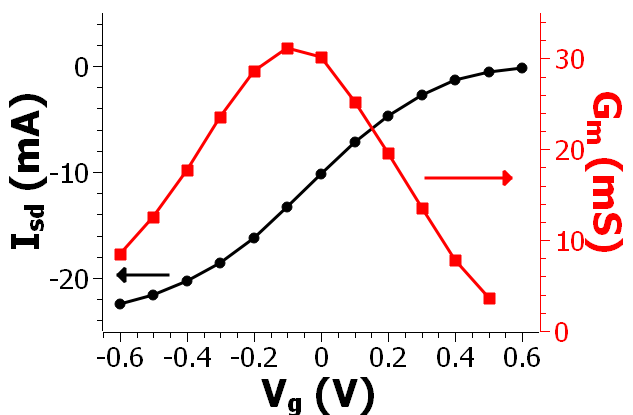


**Figure S21. Transfer characteristic of OECT.** Transfer curve (black) measured by setting source-drain voltage of -600 mV while sweeping gate voltage from -600 mV to 600 mV. Transconductance (Gm) was obtained by taking the derivative of the transfer curve.

**5.2 EFC powers the gate of OECT**.

**5.2.1 OECT coupling with the EFC.** We connected the source measure meter (SMU) to power the source-drain channel, as shown in Figure S22. The OECT was held at a constant *V_sd_* of – 600 mV while monitoring the *I_sd_.* We let the current stabilize for 50 seconds before connecting the cathode to the gate of OECT. After *I_sd_* reached a steady state, 1 M glucose solution was spiked to the anodic chamber over time. The current was constantly monitored, as shown in Fig. S23.


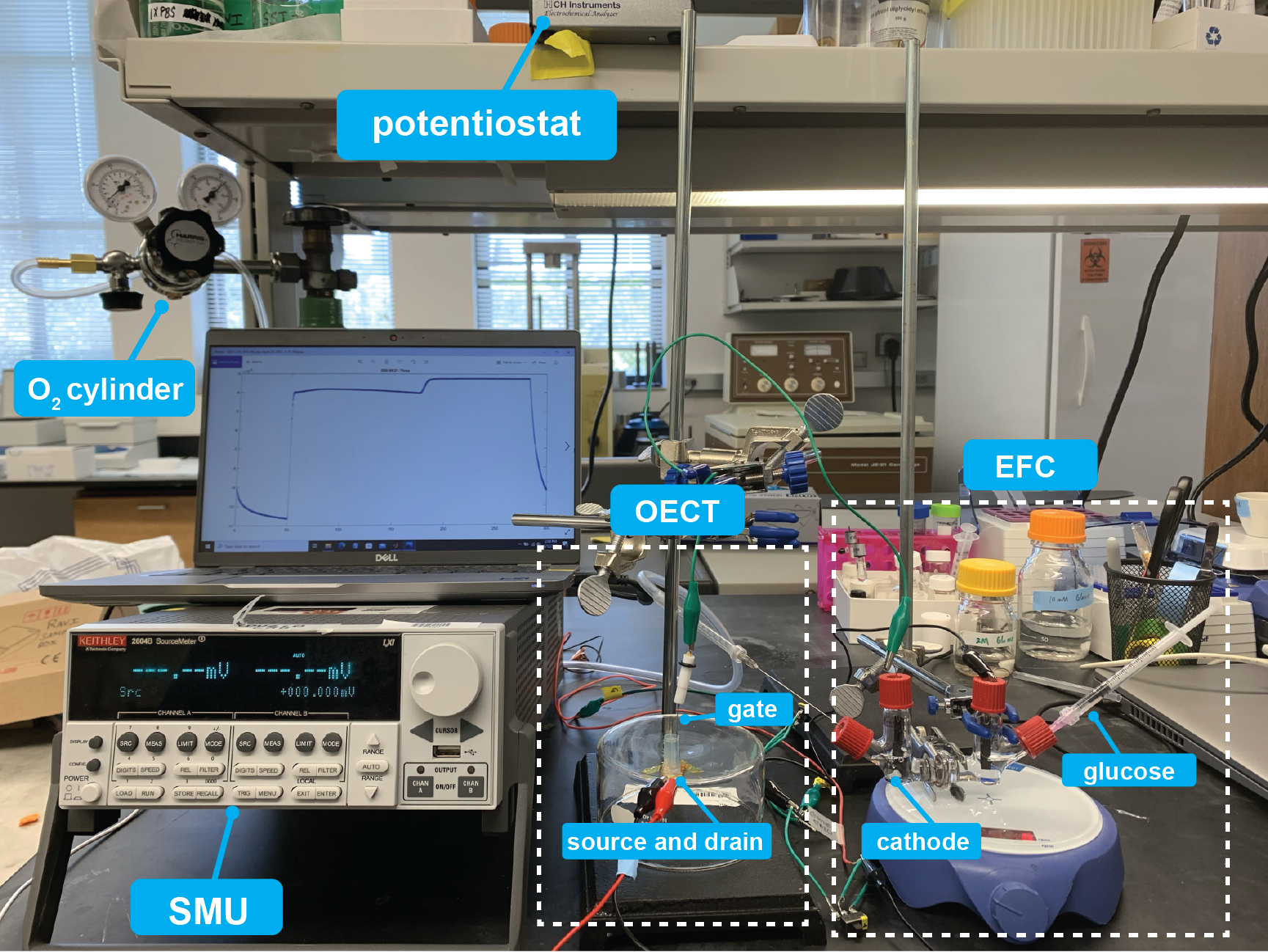


**Figure S22. A photo of the OECT coupling with EFC through the gate.**


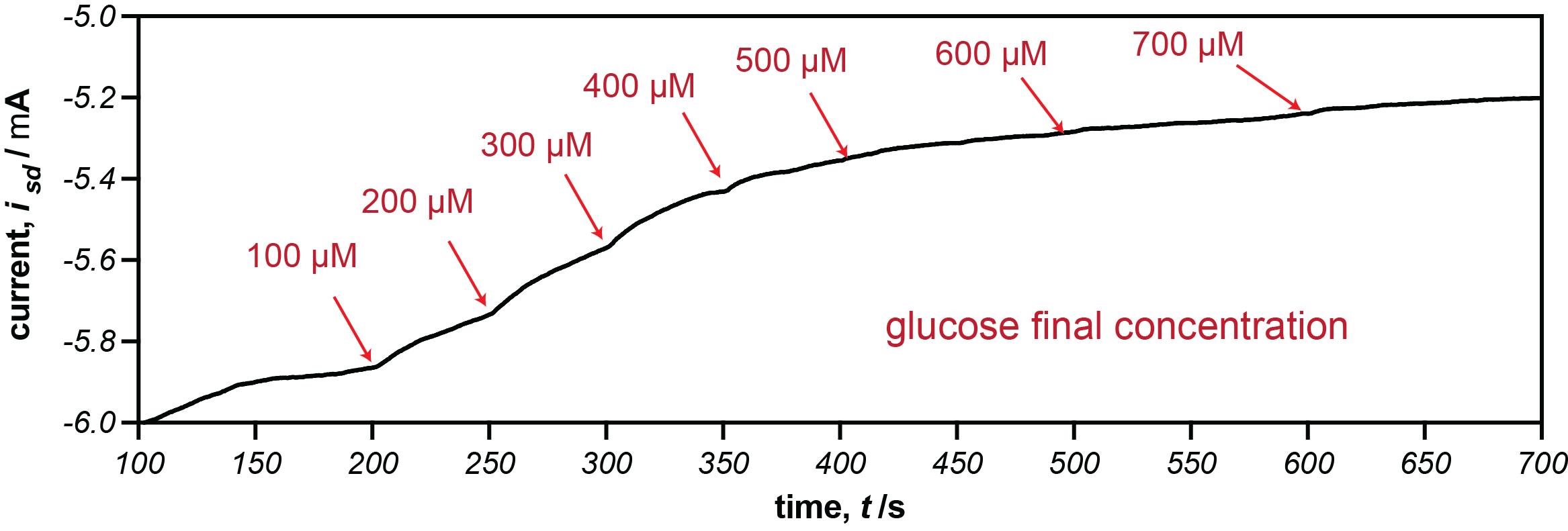


**Figure S23. A typical *I_sd_* vs. time response to glucose.** OECT was held at a constant V_sd_ of – 600 mV. 1 M glucose solution was spiked to the anodic chamber over time. The values shown in the graph indicate the final concentration of glucose in the anodic chamber (n=1).

**5.3 Mathematical model of self-powered sensor coupled to OECT.**

Here, we present a mathematical description of the time-dependence of gate voltage in the self-powered sensor coupled to OECT. We do this by deriving the time-dependent open circuit potential (OCP) for an enzymatic fuel cell (EFC) and then relating the time-dependent OCP to the drain current (*I_sd_*) of an OECT.

First, we derive a mathematical description of the time-dependent open circuit potential (OCP) for an enzymatic fuel cell (EFC) by combining the thermodynamic framework for the electrochemical cell potential with the kinetic description of enzyme-catalyzed reactions. From a thermodynamic perspective, the OCP of the EFC is given by the difference between the electrode potential of the cathode and anode:

$\mathrm{OCP}=E_{cathode}-E_{anode}$ (5)

For a reversible half-reaction reaction, the electrode potential $(E)$ is related with the formal standard potential$(E{^{\circ}}^{'})$ of the redox species and the relative concentration of the oxidized (Ox) and reduced (Red) forms, according to the Nernst equation

$E=E{^{\circ}}^{'}+\frac{RT}{nF}ln\frac{[Ox]}{[Red]}$ (6)

where $n$is the number of electrons transferred in the redox event,$R$ is the gas constant in J K^-1^ mol^-1^, $T$ is temperature in K and $F$is Faraday constant 96480 C mol^-1^.

Substituting the expressions for the electrode potentials (Eqn 6) into Eqn 5, we can write the following expression for OCP

$\mathrm{OCP}=\left( E_{\frac{O_{2}}{H_{2}O}}^{{^{\circ}}^{'}}+\frac{RT}{4F}ln\frac{C_{O_{2}}^{*}}{C_{H_{2}O}^{*}} \right)-\left( E_{\frac{gluconolactone}{glucose}}^{{^{\circ}}^{'}}+\frac{RT}{2F}ln\frac{C_{gluconolactone}^{*}}{C_{glucose}^{*}} \right)$ (7)

where $C^{*}$ is the bulk concentration of redox species.

Assuming that the concentrations of oxygen and water remain constant, we can simplify Equation 7 as

$\mathrm{OCP}=K_{total}+\frac{RT}{2F}ln\frac{C_{glucose}^{*}}{C_{gluconolactone}^{*}}$ (8)

where $K_{total}$=$E_{\frac{O_{2}}{H_{2}O}}^{{^{\circ}}^{'}}+ \frac{RT}{4F}( \frac{C_{O_{2}}^{*}}{C_{H_{2}O}^{*}})- E_{\frac{gluconolactone}{glucose}}^{{^{\circ}}^{'}}$

The concentration of glucose and gluconolactone will change due to enzymatic activity of GDH-5E^+^. Experimentally, we observe that GDH-5E^+^ kinetics could be described by the Michaelis-Menten framework, so we use this framework to describe the time evolution of the concentration of glucose and gluconolactone. For a given concentration of GDH-5E^+^, the initial velocity of this enzyme-catalyzed reaction is constant, so that the glucose and gluconolactone concentrations can be written as:

$C_{glucose}^{*}\left( t \right)=C_{glucose}^{0}-v_{i}t$ (9)

$C_{gluconolactone}^{*}\left( t \right)=v_{i}t$ (10)

where the initial glucose concentration is $C_{glucose}^{0}$, and gluconolactone concentration is 0. The initial velocity is described by Michaelis-Menten equation:

$v_{i}=\frac{k_{cat}C_{enzyme}^{0}C_{glucose}^{0}}{K_{M}+C_{glucose}^{0}}$ (11)

At high concentrations of glucose, $K_{M}+C_{glucose}^{0}\approx C_{glucose}^{0}$, so $v_{i}=k_{cat}C_{enzyme}^{0}$

Introducing this expression for initial velocity into Eqns 9,10 yields:

$C_{glucose}^{*}\left( t \right)=C_{glucose}^{0}-k_{cat}C_{enzyme}t$ (12)

$C_{gluconolactone}^{*}\left( t \right)=k_{cat}C_{enzyme}t$ (13)

Substituting these expressions for $C_{glucose}^{*}\left( t \right)$ and $C_{gluconolactone}^{*}\left( t \right)$ into Eqn 8 leads to the following equation for OCP change over time:

$\mathrm{OCP}\left( t \right)= K_{total}+ \frac{RT}{2F}ln\frac{C_{glucose}^{0}-k_{cat}C_{enzyme}t}{k_{cat}C_{enzyme}t}$ (14)

Since ${V_{max}=k}_{cat}C_{enzyme}$, we can rewrite the above equation as

$\mathrm{OCP}\left( t \right)=K_{total}+\frac{RT}{2F}ln\left( \frac{C_{glucose}^{0}-V_{max}t}{V_{max}t} \right)$ (15)

Thus, Equation 15 describes the time-dependence of the OCP for a GDH-5E^+^driven EFC.

Next, we relate the time-dependent OCP to the drain current within a coupled EFC-OECT device. Following the work of Inal *et al.* on EFC-OECT integrated glucose sensors, the glucose concentration dependent OCP controls the gate voltage (*Vg*) to dictate the drain current (*I_sd_*) ^10^

$\frac{dI_{sd}}{dt}\propto\frac{dOCP}{dt}$ (16)

By differentiating Eqn 15 with respect to time, we can determine $\frac{dOCP}{dt}$

$\frac{dOCP}{dt}=\frac{RT}{2F}\left( \frac{-V_{max}}{C_{glucose}^{0}-V_{max}t}-\frac{1}{t} \right)$ (17)

Therefore, we correlated V_max_ to $\frac{dOCP}{dt}$

|$\frac{dOCP}{dt}\vert\propto\vert\frac{-V_{max}}{C_{glucose}^{0}-V_{max}t}\vert$ (18)

As previous study, OCP controls the gate voltage (*Vg*) to dictate the drain current (*I_sd_*), therefore

|$\frac{dI_{sd}}{dt}\vert\propto\vert\frac{-V_{max}}{C_{glucose}^{0}-V_{max}t}\vert$ (19)

**6. Supplementary References**

1. W. Coyote-Maestas, D. Nedrud, S. Okorafor, Y. He, D. Schmidt, Targeted insertional mutagenesis libraries for deep domain insertion profiling. *Nucleic acids research* **48**, e11-e11 (2020).

2. A. D. Bosley, M. Ostermeier, Mathematical expressions useful in the construction, description and evaluation of protein libraries. *Biomolecular engineering* **22**, 57-61 (2005).

3. O. Salazar, L. Sun, in *Directed enzyme evolution*. (Springer, 2003), pp. 85-97.

4. A. J. Olsthoorn, J. A. Duine, Production, Characterization, and Reconstitution of Recombinant Quinoprotein Glucose Dehydrogenase (Soluble Type; EC 1.1. 99.17) Apoenzyme ofAcinetobacter calcoaceticus. *Archives of biochemistry and biophysics* **336**, 42-48 (1996).

5. M. T. Meredith, D. P. Hickey, J. P. Redemann, D. W. Schmidtke, D. T. Glatzhofer, Effects of ferrocene methylation on ferrocene-modified linear poly(ethylenimine) bioanodes. *Electrochimica Acta* **92**, 226-235 (2013).

6. D. P. Hickey, Ferrocene-Modified Linear Poly (ethylenimine) for Enzymatic Immobilization and Electron Mediation. *Enzyme Stabilization and Immobilization: Methods and Protocols*, 181-191 (2017).

7. M. T. Meredith, S. D. Minteer, Biofuel cells: enhanced enzymatic bioelectrocatalysis. *Annual review of analytical chemistry* **5**, 157-179 (2012).

8. R. Cai *et al.*, Confocal Raman Microscopy for the Determination of Protein and Quaternary Ammonium Ion Loadings in Biocatalytic Membranes for Electrochemical Energy Conversion and Storage. *Analytical chemistry* **89**, 13290-13298 (2017).

9. D. P. Hickey *et al.*, Pyrene Hydrogel for Promoting Direct Bioelectrochemistry: ATP-Independent Electroenzymatic Reduction of N2. *Chemical Science*, (2018).

10. Ohayon, D. et al. Biofuel powered glucose detection in bodily fluids with an n-type conjugated polymer. *Nature materials* **19**, 456-463 (2020).
