# Supplementary material for: Creation of a point-of-care therapeutics sensor using protein engineering, electrochemical sensing and electronic integration
Source: Nat Commun. 2024 Feb 24;15:1689. doi: 10.1038/s41467-024-45789-9 (PMC11258353; doi:10.1038/s41467-024-45789-9)
Supplement: Supplementary file 3 — Description of Additional Supplementary Files [file 41467_2024_45789_MOESM3_ESM.pdf]

Description of Additional Supplementary Files

Supplementary Data 1. Strains, plasmids, heterologous genes, and oligonucleotides used in this study.
